# Cytotoxic Autophagy: A Novel Treatment Paradigm against Breast Cancer Using Oleanolic Acid and Ursolic Acid

**DOI:** 10.3390/cancers16193367

**Published:** 2024-10-01

**Authors:** Kunj Bihari Gupta, Jie Gao, Xin Li, Muthusamy Thangaraju, Siva S. Panda, Bal L. Lokeshwar

**Affiliations:** 1 Georgia Cancer Center, Augusta University, Augusta, GA 30912, USA; kugupta@augusta.edu (K.B.G.); gaoj@uab.edu (J.G.); xli@cau.edu (X.L.); 2 Department of Clinical and Diagnostic Science, University of Alabama at Birmingham, Birmingham, AL 35233, USA; 3 The Center for Cancer Research and Therapeutic Development, Clark Atlanta University, Atlanta, GA 30314, USA; 4 Department of Biochemistry and Molecular Biology, Medical College of Georgia, Augusta University, Augusta, GA 30912, USA; mthangaraju@augusta.edu (M.T.); sipanda@augusta.edu (S.S.P.); 5 Department of Chemistry and Biochemistry, College of Science and Mathematics, Augusta University, Augusta, GA 30912, USA

**Keywords:** bioactive triterpenoids, triple-negative breast cancer, PI3 kinase inhibition, mitophagy, combination therapy

## Abstract

**Simple Summary:**

Cancer chemotherapy is dominated by cytotoxic drugs that non-selectively kill proliferating cells by blocking DNA replication and inducing apoptosis. Some natural anticancer products are less toxic to normal, slow-replicating cells but kill aggressive tumor cells. The combination of two structural isomers of pentacyclic triterpenes, oleanolic acid and ursolic acid, showed potent cytotoxic activity in human breast cancer cells in vitro by inducing cytotoxic autophagy at a significantly lower dose than previously reported. They were nontoxic to breast epithelial cells. Their activity was mainly due to the inhibition of AKT-mediated cell survival and the inhibition of the mammalian target of rapamycin (mTOR) signaling pathway, a central regulator of cell metabolism, growth, and proliferation. The compounds were equally effective against ER+ and triple-negative breast cancer cells. Combining low doses of nontoxic inhibitors of PI3k kinase increased the activities of these compounds. In contrast, inhibition of autophagy by 3-methyl adenine annulled their activity, demonstrating that cytotoxic autophagy may be the dominant mechanism of cell death. These compounds have potential for cancer therapy in neoadjuvant or adjuvant settings.

**Abstract:**

Background: Oleanolic acid (OA) and Ursolic acid (UA) are bioactive triterpenoids. Reported activities vary with the dose used for testing their activities in vitro. Studies using doses of ≥20 µM showed apoptosis activities in cancer cells. However, reported drug levels in circulation achieved by oral administration of UA and OA are ≤2 µM, thus limiting their use for treatment or delivering a combination treatment. Materials and Methods: The present report demonstrates the efficacy of OA, UA, and OA + UA on tumor cell-specific cytotoxicity at low doses (5 µM to 10 µM) in breast cancer (BrCa) cell lines MCF7 and MDA-MB231. Results: The data show that both OA and UA killed BrCa cells at low doses, but were significantly less toxic to MCF-12A, a non-tumorigenic cell line. Moreover, OA + UA at ≤10 µM was lethal to BrCa cells. Mechanistic studies unraveled the significant absence of apoptosis, but their cytotoxicity was due to the induction of excessive autophagy at a OA + UA dose of 5 µM each. A link to drug-induced cytotoxic autophagy was established by demonstrating a lack of their cytotoxicity by silencing the autophagy-targeting genes (ATGs), which prevented OA-, UA-, or OA + UA-induced cell death. Further, UA or OA + UA treatment of BrCa cells caused an inhibition of PI3 kinase-mediated phosphorylation of Akt/mTOR, the key pathways that regulate cancer cell survival, metabolism, and proliferation. Discussion: Combinations of a PI3K inhibitor (LY294002) with OA, UA, or OA + UA synergistically inhibited BrCa cell survival. Therefore, the dominance of cytotoxic autophagy by inhibiting PI3K-mediated autophagy may be the primary mechanism of PTT-induced anticancer activity in BrCa cells. Conclusion: These results suggest it would be worthwhile testing combined OA and UA in clinical settings.

## 1. Introduction

Globally, breast cancer (BrCa) ranks second in incidence and mortality among women with cancer. The American Cancer Society reports about 310,720 women are likely to be diagnosed with invasive BrCa, with an estimated 42,250 deaths in the USA [1]. These grim data portray a dire situation where, despite the multiple modalities of treatment and therapy with multiple anticancer drugs approved by agencies such as the FDA, BrCa is a significant health concern in women. More potent, yet less toxic treatment must be developed for therapies against BrCa.

The patient’s BrCa treatment depends on the patients’ tumor stage and age [2]. The common initial treatment for localized disease is surgery or non-invasive high-beam radiation therapy. The non-surgical or post-surgical treatment options for BrCa patients vary, but most rely on cytotoxic chemotherapy drugs for post-surgical or radiation treatment to eliminate residual tumor cells in lymph node-positive BrCa patients. However, many women who receive adjuvant chemotherapy for early-stage breast cancer do not benefit significantly [3]. The cytotoxic chemotherapeutic (drugs) can cause systemic toxicity due to non-selective targets and the emergence of more aggressive drug-resistant BrCa that further require multiple treatment regimens. Chemo-hormonal therapies are increasingly used to combat disease progression and prolong therapeutic efficacy in patients with tumor biopsies showing cells with estrogen receptor (ER), progesterone receptor (PR), and human EGF receptor 2 (HER2) positivity [4,5]. However, patients with disease progression to the ER, PR, and HER2-negative (triple receptor-negative) stage of BrCa (TNBC) are given broader options, but with limited efficacy. TNBC is generally a disseminated disease with limited access to targeting specific therapy (e.g., metastasis to brain and bones) and highly aggressive proliferation.

Recently, more specific, molecularly targeted drugs have been developed that block specific tumor cell survival pathways, such as mTOR-mediated pathway inhibitors [6,7], targeting the cell cycle and metabolism [8,9], and those affecting the tumor microenvironment, such as immunotherapies. Treatment efficacy with all these agents suffers the same fate as chemotherapy drugs, namely, progression of therapy-resistant disease or significant systemic toxicity experienced by a sizable fraction of patients who opt for immunotherapies [10,11]. Anticancer agents that enhance cytotoxic drugs’ therapeutic efficacy without significantly increasing systemic toxicity should improve the effectiveness and therapeutic index. Synthetic inhibitors of critical signaling molecules that trigger tumor cell proliferation, metastasis, and development of resistance have been extensively developed, yet much is needed to eliminate cancer deaths. Bioactive phytochemicals, identified by serendipity or empirical research, have been constantly evolving and tested for potential application in BrCa treatment. Several candidate phytochemicals have been investigated to treat BrCa, including many forms of curcumin, triterpenes, antiestrogens (e.g., genistein), polyphenols, and others. However, most of these compounds are seldom used in the clinic, as they do not meet the rigors of sufficient clinical efficacy [12]. However, natural compounds with unique bioactivities as anti-inflammatory and antiproliferative agents may offer significant advantages in treating patients alone or in a neoadjuvant or adjuvant setting.

The pentacyclic triterpenoids, such as ursolic acid (UA) and oleanolic acid (OA), are triterpenoids that function similarly to plant steroid hormones that are bioactive in human cells [13]. These compounds and betulinic acid are the most studied among triterpenes for human health [14,15]. Due to their abundance and ubiquitous presence in many edible plant products, UA and OA have been extensively investigated for their bioactivities in multiple ailments, including cancer [16,17,18], and their mechanisms of action in cancer cells [19,20]. However, in nature, OA and UA exist as structural isomers and their yields vary extensively from various natural sources, such as olives and apple peels [21,22]. Despite the molecular equivalency, there have been studies on a combination of OA and UA against cancer cells [23,24,25,26]. We saw significant potential for combining OA and UA to enhance the bioactivity, as both compounds have shown minimum systemic toxicity in the limited toxicological studies in humans [27]. In this study, we investigated the bioactivity and mechanism of OA and UA individually and in combination at various ratios to identify any advantage of using a specific combination of OA and UA to enhance bioactivity in BrCa cells. We report that the combination of OA and UA shows higher cancer cell-specific toxicity at lower doses than when used individually.

## 2. Materials and Methods

### 2.1. Chemicals of Certified Highest Quality from the Manufacturers Used in This Study

They were purchased from laboratory suppliers based in the USA, and details of these specific chemicals and kits are listed below in Table 1.

### 2.2. Cell Culture Methods

The human BrCa cell lines MCF7 and MDA-MB231 and non-malignant breast epithelial cell line MCF-12A were purchased from the ATCC (Manassas, VA, USA). All studies reported here were from cell cultures derived from aliquots frozen during the first five passages of ATCC stocks, as described before [28]. Periodically, cells were authenticated by Lab Corp Cell Line Authentication Lab, Burlington, NC. All cell lines were routinely cultured in the media recommended by ATCC (ATCC.Org, Manassas, VA, USA). Briefly, we maintained cells in RPMI 1640 medium supplemented with fetal bovine serum (10%, Bio-West USA Inc., Bradenton, FL, USA) and 0.2 mg/L gentamycin solution (IBI Scientific, Dubuque, IA, USA) with passaging every 5–7 days. All drug treatments were performed in cells cultured in multiwell plates with a seeding density of ~3–6 × 10^3^ cells/cm^2^ or as indicated for each experiment. Cultures were maintained in water-jacketed incubators with sterile air with 5% CO_2_ and maintained at 37 °C.

### 2.3. Cell Viability Assay

The viability of cells treated with several doses of OA, UA, and their combination were estimated using a colorimetric assay, the 3-[4,5-dimethylthiazol-2-yl]-2,5-diphenyl-tetrazolium bromide (MTT) reduction assay. Briefly, cells were cultured in 96-well plates at a density of 2000 cells per well, and overnight-cultured cells were treated with the appropriate doses of OA, UA, and both for 72 h. Following incubation, the viability of surviving cells was determined by incubating with MTT solution (0.5 mg/mL) at 37 °C for three hours. The precipitated purple formazan crystals were dissolved in DMSO, and absorbance was read at 570/640 nm with a spectrophotometer. The fraction of viable cells is expressed as a percentage of viable cells from the ratio of the OD of treated wells divided by the OD of diluent-only treated cultures (control) [29,30].

### 2.4. Clonogenic Survival (Colony-Forming) Assay

The BrCa cells were cultured in replicate 35 mm-diameter 6-well plates at a density of ~1000 cells per well. The cell cultures were incubated with various doses of OA, UA, and both for 72 h. The culture medium was changed to remove drugs, and surviving cells were left to form colonies for the next 7–10 days. Emerged cell colonies after incubation were fixed with methanol and stained with 0.5% crystal violet for 30 min. Then, they were washed gently to remove excess dye solution and air-dried before manually counting the number of visible colonies by at least two investigators [31].

### 2.5. Target Identification by Immunoblotting

We prepared cell lysates from cultured cells treated with several doses of OA and UA and estimated total proteins in the lysates. Required sample lysates were boiled in SDS–polyacrylamide gel electrophoresis (SDS-PAGE) sample buffer, fractionated by SDS-PAGE under chemically reduced conditions, and blotted onto PVDF membranes for Western blot analysis as reported before [32]. The blots were incubated with indicated primary and secondary antibodies (see Table 2) and developed to visualize protein bands using an enhanced chemiluminescence detection kit [31] Blot images were captured using the Bio-Rad ChemiDoc™ Touch imaging system. Unless indicated otherwise, blots of β-actin in each sample were used as an internal loading control [31].

### 2.6. Apoptotic DNA Fragmentation Assay: TUNEL Assay

Cells undergoing apoptotic death were detected by fluorescence labeling of their DNA fragments using a terminal deoxynucleotidyl transferase dUTP nick-end labeling kit (TUNEL kit, Table 1) according to the manufacturer’s instructions. Briefly, cells were cultured and treated with 0.05% DMSO (Control), 2.0 µM staurosporine as a positive control (Sigma Aldrich Inc., St. Louis, MO, USA), OA, UA, and both for 48 h. Treated cells were fixed with 4% paraformaldehyde (PFA) and permeabilized with 0.1% Triton X-100 solution. The intracellular fragments of DNA were then labeled with TUNEL reaction mixture for 1 h at 37 °C in a humidified chamber in the dark. Labeled cells in culture chambers were washed twice and mounted with a coverslip before microscopy. The fluorescence images of labeled cells were captured using a Keyence inverted fluorescence microscope [33].

### 2.7. Localization of LC3B and LC3BII by Immunofluorescence

Subcellular localization and formation of LC3B puncta were examined by fluorescence microscopy of cells labeled with anti-LC3BII antibody and anti-Rb-Alexa fluor IgG-Fab2 and counterstained with DAPI [4]. Slides were observed, and the fluorescence images were captured on the Keyence fluorescence microscope.

### 2.8. Autophagy Flux Determination

We used a dual-label fluorescence quench assay with cells transfected with a plasmid containing mCherry-GFP-LC3B genes in tandem sequence (Addgene Inc., Cambridge MA, USA), as reported in previous studies [4,34]. Briefly, cells were seeded in 35 mm culture dishes with glass coverslips placed in the bottom (MatTek catalogue number P35G) in an antibiotic-free media. Cultured cells were transfected with mCherry-GFP-LC3B plasmid using Lipofectamine™ 2000 (Life Technologies) as per the provider’s instructions. Transfected cells were used for subsequent treatment with drugs 48 h after transfection. After completion of treatment (24 h), cells were labeled with DAPI DNA-binding dye and observed under the fluorescence microscope without formalin fixation. Fluorescence images of live cells were captured.

### 2.9. Alteration of ATG5 and ATG7 Levels Using siRNA-Mediated Gene Silencing

Human-specific small interfering RNA (siRNA) for ATG5 and ATG7 and scrambled siRNA were purchased from a vendor (Dharmacon, Thermo Fisher Scientific Inc., Waltham, MA, USA). The MCF7 andMDA-MB231 cells were cultured in 6-well plates and transfected with siRNAs using Lipofectamine™ 2000. Transfected cells were treated with UA, OA, and both for 48 h. Treated and control cultures with or without specific ATG siRNA were harvested to make cell lysates and assess viability following OA and UA treatment. Protein expression for ATG5 and ATG7 in control siRNA and specific siRNA transfectants was determined by Western blotting. An MTT-reduction assay was used to estimate cell viability after silencing these genes.

### 2.10. Statistical Analysis

All quantitative assays were conducted with triplicate samples in each experiment, and the experiments were repeated at least three times. Data are presented as means ± SD from the repeated experiments. We used Student’s *t*-test or one-way ANOVA to determine statistical significance for all quantitative experiments. The comparison of combined data with a value of *p* < 0.05 against the null hypothesis was considered statistically significant. GraphPad Prism 9 (GraphPad Inc., San Diego, CA, USA) program was used for rapid analyses.

## 3. Results

### 3.1. OA and UA Specifically Inhibit the Growth and Colony-Forming Capacity of BrCa Cells, but Are Nontoxic to Normal Breast Epithelial Cells

**Figure 1:**
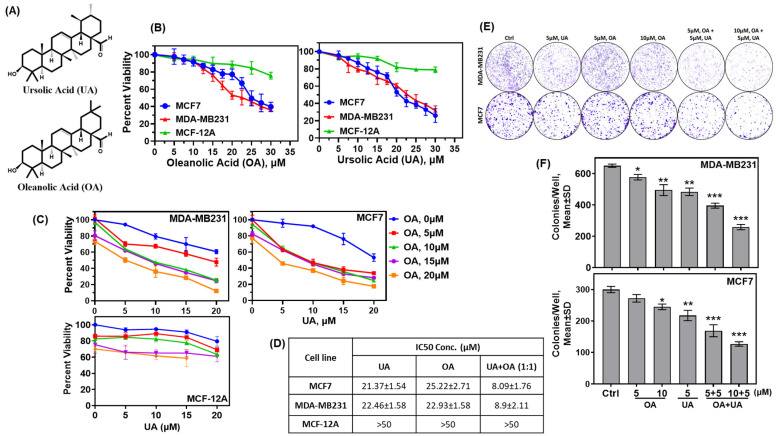
Pentacyclic triterpenes (PTTs) UA and OA induced cytotoxicity in normal breast epithelial cells and BrCa cells. (**A**) Chemical structure of UA and OA. (**B**) Cytotoxicity of OA and UA on MCF-12A (normal mammary epithelial cell line), MCF7, and MDA-MB231 cells. Surviving fractions after 72 h incubation with OA (**left** panel) and UA (**right** panel) were estimated by MTT-reduction colorimetric assay. (**C**) Surviving fractions (%) of BrCa cells and MCF-12A cells at various combinations of OA and UA. (**D**) Summary of IC50 of OA and UA on BrCa and MCF-12A cells. (**E**) Inhibition of clonogenic potential of BrCa cells treated with OA, UA, and both: colonies of surviving cells formed in 7 to 10 days following treatment with UA, OA, and both for three days. (**F**) Quantitative analysis of colonies/well (mean ± SD, n = 3) for MDA-MB231 and MCF7 cells. Pairwise *t*-tests were used to determine significance: * *p* < 0.05, ** *p* < 0.005, or *** *p* < 0.001 with untreated cell colonies as control.

Established BrCa cell lines and normal breast epithelial cells were treated with multiple doses of UA and OA separately and in combination to determine their effect on the viability of the incubated cells. The surviving cell fractions following 72 h incubation with the drugs were determined by MTT colorimetric assay, and the dose required to reduce the surviving fractions by 50% (IC50) was calculated. The IC50 of UA, OA, and OA + UA combination varied widely between the drugs and the cells tested. As listed in Figure 1A, OA and UA were weakly toxic to the non-tumorigenic MCF12A cells (IC50 > 50 µM), whereas they were toxic to tumorigenic MCF7 and MDA-MB231 cell lines. However, when cells were treated with UA and OA in a 1:1 ratio, the viability of MCF7 and MB231 cells significantly decreased, although this combination was weakly toxic to MCF-12A cells (Figure 1B).

Colorimetric assays to determine cytotoxicity using compounds such as MTT do not distinguish between the cytostatic versus cytotoxic effect of a drug or a drug’s potential to diminish the clonogenic potential of the tumor cells if the cells are cultured in the absence of drugs following treatment. We opted to evaluate the cytotoxicity of OA, UA, and OA + UA combinations following a 72 h treatment rather than the commonly used 48 h, as the tested cell lines, including the non-transformed MCF-12A, had different rates of population doubling compared to MDA-MB231. Clonogenic survival or colony-formation assays provide a more accurate determination of tumor cells’ survival potential, even after termination of therapy. We determined the activity of OA and UA and their combination to destroy the colony-forming potential without the drug. Cells were incubated with OA and UA at several doses alone or in combination for three days, followed by incubation in a drug-free growth medium for the next 7 to 10 days or until the surviving cells formed visible colonies in the culture dishes. Results of colony counts from each treatment group are shown in Figure 1B,D, showing a significant decline in colony numbers in UA- and OA-treated groups. As estimated by MTT assays in Figure 1A, colony numbers declined at all doses of UA and OA, but were significantly higher in the OA + UA treatment (Figure 1D). These results demonstrate UA- and OA-induced cytotoxicity, but not reversible cytostatic activity.

We determined whether the cytotoxic effects of combination OA and UA treatment were additive, synergistic, or non-additive by the combination effect equation of Chou and Talalay [35]. The analysis performed using the program CompusSyn (PD Science, Paramus, NJ, USA) showed the combination index (C.I) < 1.0 in all combinations of OA and UA on both BrCa cells (Supplementary Figure S1). The normalized isobologram for the OA + UA combination was <1.0 at all doses tested. These results indicate the potential to increase the efficacy of UA and OA in small combinations and reduce the dose of either drug for treatment without affecting the efficacy.

### 3.2. OA and UA Do Not Induce Programmed Cell Death (Apoptosis) in BrCa Cells

**Figure 2:**
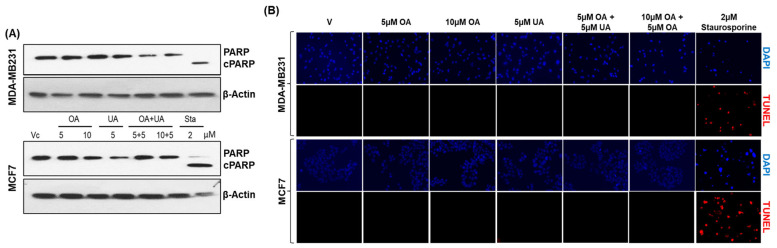
Lack of apoptotic activity in OA- and UA-treated BrCa cells. (**A**). Apoptotic activity visualized as changes in cleaved PARP by immunoblotting analysis showed a lack of cleaved PARP (c-PARP) in MDA-MB231 and MCF7 cells treated with OA, UA, and both. However, cells treated with 2 µM staurosporine (Sta) showed cPARP. β-actin bands reference the uniform loading of cell lysates in all electrophoretic blot lanes. Original images of western blots are shown in Supplementary Materials File S1. (**B**). DNA fragmentation levels in situ due to apoptosis were analyzed in BrCa cells by the TUNEL (terminal deoxynucleotidyl transferase dUTP nick-end labeling) assay. DNA fragmentation fluoresced red, and cells with intact nuclei did not. Cells treated with PTTs did not fluoresce, but those treated with staurosporine did, indicating an absence of apoptosis in PTT-treated cell cultures (Magnification: 100×).

Multiple publications have reported apoptosis as the principal mechanism of cell death in UA-treated cells. However, these studies used high doses of UA (>25 µM) in such demonstrations. Since we observed significant lethality of UA on BrCa cells at <10 µM of UA, we investigated whether apoptosis is the major mechanism of cell death at low doses of UA. We determined cleaved poly-adenosine ribose phosphorylase (PARP) levels and cellular DNA fragmentation (TUNEL) levels as markers of apoptosis in the control and PPT-treated cells. We attempted to detect both intact (116 kDa) and cleaved (89 kDa) PARP proteins in cells treated with PPTs and untreated control cells by Western blotting (Figure 2A). We found cleaved fragments of PARP only in cell lysates prepared from cells treated with the protein kinase C inhibitor staurosporine (Sta, 2.0 µM) for the same duration as other drugs in both MCF7 and MB231 cells (Figure 2A). The intact status of PARP detected in Western blots showed a lack of significant apoptosis.

Next, we examined the presence of DNA fragmentation in OA- and UA-treated cells. We used a fluorescence in situ terminal deoxynucleotidyl transferase dUTP nick-end labeling (TUNEL) assay with Texas red-labeled uridine nucleotide mix [36]. As shown in Figure 2B, Texas red-labeled TUNEL-positive fluorescence was found only in cells treated with 2 µM staurosporine, but not in cells treated with UA, OA, or UA + OA at doses ≤ 10 µM. These two results convinced us that apoptosis is not a likely cell death mechanism in BrCa cells after OA and UA treatments. The potential for other forms of apoptosis, such as necroptosis or ferroptosis, was unlikely, as the TUNEL-positive cells or cleaved PARP were uniformly absent in all treated samples at the indicated drug concentrations. Next, we investigated other potential cell death mechanisms, such as cytotoxic autophagy.

### 3.3. OA- and UA-Induced Cell Death via Activation of Autophagy

**Figure 3:**
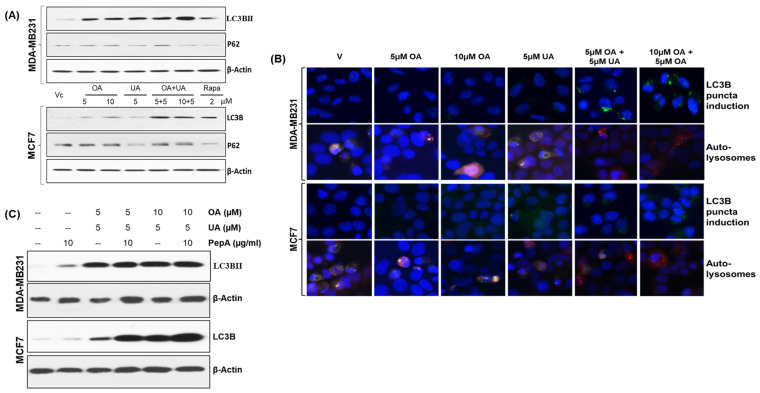
OA and UA combination treatment causes cell death in BrCa cells by cytotoxic autophagy. (**A**) Increased expression of two autophagy markers, LC3BII and P62 (SQSTM1), was found in the cells treated with OA, UA, and both in MDA-MB321 and MCF7 cells. The doses of the PTTs used for autophagy induction are shown in the middle or top panels. (**B**) The translocation of LC3B on the puncta of autophagosomes was made visible with FITC-labeled anti-rabbit antibodies against anti-LC3B antibodies, followed by counterstaining the cells with DAPI (top panel). Increased autophagic flux in PTT-treated BrCa cells was detected using differential localization of mCherry-GFP-LC3B-transfected cells treated with PTTs. The LC3B molecules exiting from neutral pH autophagosomes to acidic autolysosomes are shown. An increased level of autolysosomes in treated cells visualized by red fluorescence (mCherry) and quenching of GFP (green) in the acidic compartment of autophagosomes resulting from the fusion of autophagosomes and lysosomes. (**C**). Western blot analysis of LC3BII protein after treating both BrCa cell lines with OA, UA, and both with and without PepA (pepstatin A, an inhibitor of lysosomal proteases). The micrographs (magnification: 200×) shown are representative images from three replicate experiments. Original images of western blots are shown in Supplementary Materials File S1.

Several researchers have reported autophagy as a potential cell death mechanism. It has been reported that UA and OA induce autophagy in BrCa cells [14], where their cytotoxicity may be attributed to both apoptosis and autophagy. We determined the levels of the LC-3B protein (microtubule-associated protein 1A/1B-light chain 3 (LC3)) and P62/SQSTM1, well-known autophagy markers. As shown in Figure 3A, the levels of LC3B and P62 expression increased in the cells upon treatment with OA, UA, their combination, and rapamycin (a known inducer of autophagy).

The expression of LC3B and its externalization as LC3BII in the puncta were observed following immunofluorescence labeling with anti-LC3B antibody. LC3BII localization in the puncta is an indicator of increased autophagic flux. The autophagosome formation seen in MCF7 and MDA-MD231 cell cultures treated with OA, UA, and OA + UA or rapamycin (Figure 3B, first and third panels) shows increased autophagy following drug treatment.

The cytoplasmic LC3B (LC3BI) is constitutively expressed, but the lighter chair, LC3BII, is associated with phagosome formation with characteristic puncta [4]. We determined the relative proportion of LC3B to LC3 using dual-autofluorescence protein labeled LC3 expression plasmid. A vital step of the autophagy process is the formation of an autolysosome. We utilized a selective in situ fluorescence quench assay to determine the dynamics of autophagy flux. For this, BrCa cells were transiently transfected with the mCherry-GFP-LC3 vector. The increase in autophagy (flux) resulted in the localization of LC3BII in acidic lysosomes, which quenched GFP. This quenching made only red (mCherry) fluorescence visible. If any drug treatment does not cause an increase in autophagy flux, the cells exhibit predominantly green fluorescence (GFP) or a mix of green and red (orange) fluorescence. As shown in Figure 3B (second panel for MDA-MB231 cells and fourth panel for MCF7 cells), a significant increase in overall autolysosomes was found, also predominantly displaying red fluorescence, indicating increased flux and thus rapid recycling of degraded proteins for catabolism. The flux was further enhanced in MDA-MB231 and MCF7 cells upon exposure to OA + UA. The autolysosome formation and red fluorescence were comparatively high in cells with OA + UA combination treatment.

We confirmed the increased occurrence of autophagy following exposure to OA and UA by treating the cells (or not) with pepstatin A (PepA), a known inhibitor of lysosomal proteases. Cell lysates were analyzed by immunoblotting for LC3BII. As presented in Figure 3C, the LC3BII levels did not decrease upon treatment with PepA in OA- or UA-treated cells, indicating the increased LC3BII levels were due to increased conversion of LC3BI to the autosomal puncta. These findings strongly support the occurrence of autophagy upon exposure to OA and UA.

### 3.4. Mechanism of Increased Autophagy in OA-, UA-, and OA + UA-Treated BrCa Cells

**Figure 4:**
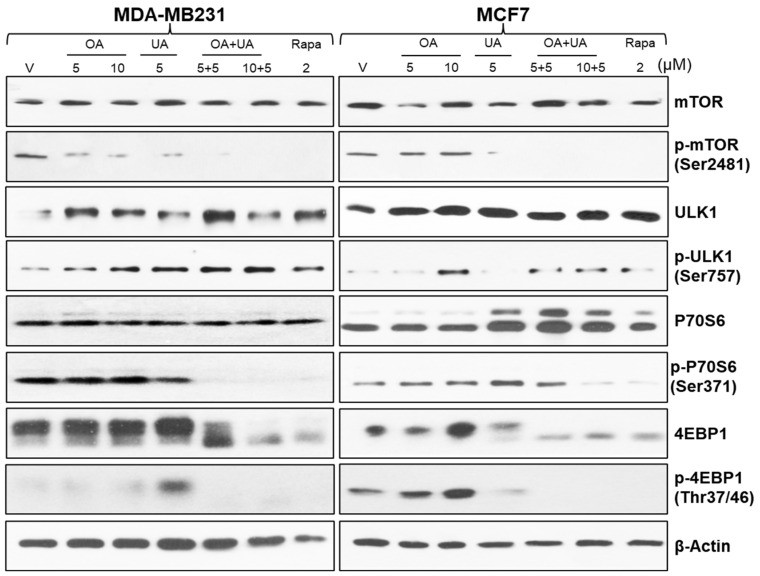
The molecular landmarks of autophagy-induced cell death in BrCa cells were analyzed by Western blotting of cell lysates from BrCa cells treated with OA, UA, and both. Molecular analysis of rapamycin-treated cells served as a positive control. β-actin was used as an internal loading control. Results were comparable from all three replicate experiments. Original images of western blots are shown in Supplementary Materials File S1.

Autophagy constitutes an intricate biological process characterized by the orchestrated involvement of diverse proteins operating sequentially. We analyzed the cell lysates prepared from BrCa cells (MCF7 and MDA-MB231) treated with several doses and a combination of OA, UA, and rapamycin. The mammalian target of rapamycin (mTOR) is a crucial regulator of signals involved with protein synthesis, cell growth, and metabolism. It is also a master regulator of autophagy [37,38]. Under a normal state, mTOR is associated with other induction complexes (ULK1 and Atg13). It is reported that inhibition of mTOR phosphorylation (phospho-mTOR, p-mTOR) induces autophagy. As shown in Figure 4, the levels of p-mTOR (p-Ser2481) were significantly reduced in UA- and OA + UA-treated cells of both MCF7 and MB-231 lines, but OA did not inhibit p-mTOR.

Interestingly, the expression of ULK1 and phospho-ULK1 levels differentially varied in MCF7 and MDA-MB231 cells. ULK1 and p-ULK1 levels were low in control (0.1% DMSO, vehicle) MDA-MB231 cells, and p-ULK1 was increased with OA, UA, and OA + UA combination treatment (Figure 4, rows 3 and 4).

Ribosomal protein S6 kinase beta 1 (P70 S6 kinase) is a crucial regulator of the cell cycle, cellular proliferation, and autophagy process. The expression of P70 S6 kinase and P70 S6 kinase (Ser371) were high in treated cells compared with untreated, but its expression was higher following UA and OA + UA combination treatments (Figure 4) in both MCF7 and MDA-MB231 cells.

Eukaryotic translation initiation factor 4E-binding protein 1 (4EBP1) is a downstream effector of mTOR and negatively regulates autophagy. 4EBP1 was constitutively expressed in vehicle-only treated cells and cells treated with OA or UA alone. Still, its expression was drastically reduced in cells treated with OA + UA combination or rapamycin. The phosphorylated form of 4EBP1, 4EBP1Thr37/46 expression was minimal in MDA-MB231 cells following treatment but was higher in MCF7 cells. These results cumulatively indicate the initiation of phagophore formation in treated cells, especially with the OA + UA combination, and progression of the autophagosome, with ultimate induction of excessive autophagy-induced cell death.

### 3.5. OA- and UA-Induced Autophagy Is Regulated by ATG5 and ATG7

**Figure 5:**
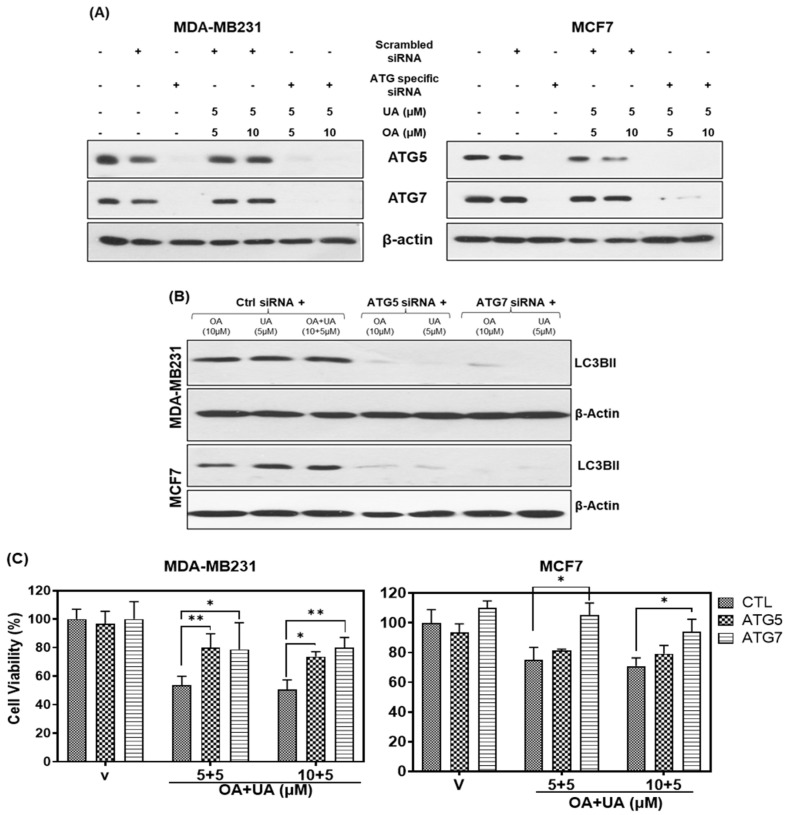
The knockdown of ATGs blocks the PTT-induced autophagy and cell death. (**A**) Immunoblotting after silencing of ATG7 and ATG5 by siRNA and treating with OA and UA in both BrCa cells (MDA-MB231 and MCF7). (**B**) Cell viability assay: increased cell viability in ATG knockdown BrCa cells compared to the control siRNA after OA and UA treatments. Results are expressed as mean ± SD (n = 3), and pairwise *t*-tests were used to determine the significance: *p* < 0.05 = * *p* < 0.005 = **. (**C**) Induction of autophagy (level of LC3BII) after treating the ATG knockdown BrCa cells with OA, UA, and both. β-actin was used as an internal loading control for all Western blots. Experiments were performed thrice with comparable results. Original images of western blots are shown in Supplementary Materials File S1.

To understand the induction of autophagy cascade resulting from OA, UA, and OA + UA treatments, we investigated the role of two critical autophagy-regulating genes (ATGs) by transiently depleting ATG7 and ATG5 in both MCF7 and MDA-MB231 cells by siRNAs and measuring the viability of depleted cells when treated with OA, UA, and OA + UA. As shown in Figure 5A, ATG5, and ATG7 proteins were completely depleted using the on-target siRNA-transfected cells, whereas their expression was unaltered in cells transfected with scrambled siRNA (control). Furthermore, treating the transfected cells with UA, OA, and OA + UA did not increase the expression of these two ATGs in mRNA-depleted MCF7 or MDA-MB231 cells. The expression of LC3BII in ATG-mRNA-depleted cells was undetectable in both cell lines, irrespective of drug treatment (Figure 5B).

We next investigated if inhibition of autophagy regulators could reduce the cytotoxicity of OA, UA, or both in the two BrCa cell lines. We performed cell viability assays on the ATG knockdown cells after treating them with OA, UA, and OA + UA. The results presented in Figure 5C show increased viability by >50% in MB231 and >40% in MCF7 cells that did not express ATGs or LC3Bs compared to scrambled siRNA (control)-transfected cells. These observations indicated that inhibiting the ATG5 and ATG7 significantly, although not wholly, rescued OA- and UA-induced cytotoxicity, further corroborating other related observations presented in this report.

### 3.6. OA and UA Inhibit AKT Signaling Pathway

**Figure 6:**
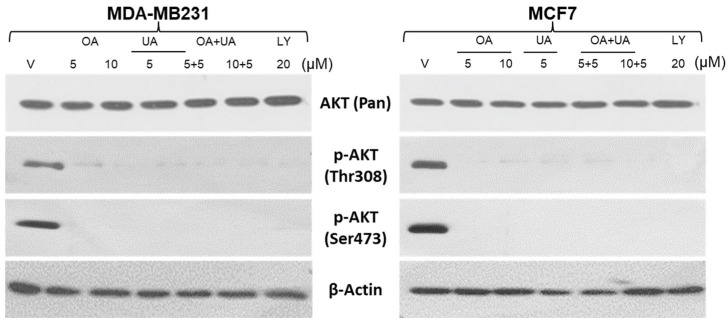
PTTs downregulate AKT activation. Western blotting analyses were performed to estimate the extent of activated AKT (phosphorylated (Thr308) and Ser473) and total AKT in whole-cell lysates of MDA-MB231 and MCF7 cells. The cells were treated with OA, UA, and both with or without LY94002 (PI3K inhibitor). β-actin is used as an internal loading control. Original images of western blots are shown in Supplementary Materials File S1.

Previous reports show that mTOR negatively regulates autophagy in a transcription-independent manner, which is tightly controlled downstream of AKT activation-induced signaling, where AKT is phosphorylated at Thr308 (by PDK1) and Ser473 (by PDK2). The AKT and phosphorylated AKT expression in both BrCa cells were examined after treating the cells with OA, UA, and OA + UA. Cell lysates were prepared and analyzed following a fixed period of drug exposure. We used a well-established inhibitor of AKT activation, LY294002 (LY; a broad-acting reversible PI3K inhibitor), as a positive control for this experiment. As shown in Figure 6, the pAKT (Thr308) level decreased after 48 h of exposure, but in the case of combination treatment, its decrease was more acute. Nearly the same trend was observed in the case of pAKT (Ser473), but its decrease in expression was more prominent than pAKT (Thr308). However, the expression of total AKT did not show a significant change as the time of exposure increased (Figure 6).

**Figure 7:**
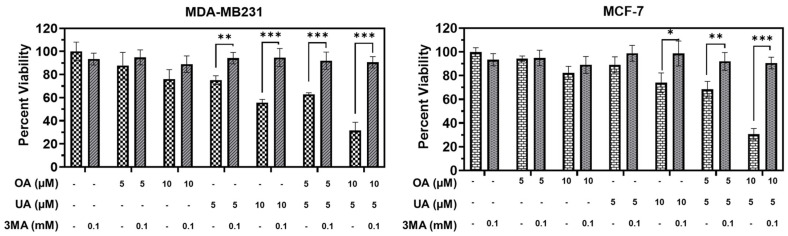
Treatment with 3-methyladenine (3MA) attenuates PTT-induced cytotoxicity. Cell viability recovered significantly following simultaneous incubation with 3MA and PTTs in MDA-MF231 and MCF7 cell cultures, indicating the cells were solely dependent on cytotoxic autophagy as PTT-induced autophagy. Results are expressed as means ± standard deviation (SD) (n = 3), and pairwise *t*-tests were used to determine significance: *p* < 0.05 = *, *p* < 0.005 = **, *p* < 0.001 = ***.

We next examined whether autophagy inhibitors would negatively regulate OA-, UA-, and OA + UA-induced autophagic death. We used a low dose of 3-methyladenine (3MA), which inhibits autophagosome formation and promotes autophagy progression. Regarding cell viability in the presence and absence of 0.1 µM 3MA and various OA, UA, and OA + UA doses, we observed that co-incubation of OA, UA, and both in the presence of 3MA nearly abolished the cytotoxic lethality of OA, UA, and OA + UA (Figure 7). Although the common dose of 3MA used in such experiments was ≥5 mM, 3MA was able to inhibit autophagy-induced cell death completely in both MDA-MB231 and MCF7 cells.

**Figure 8:**
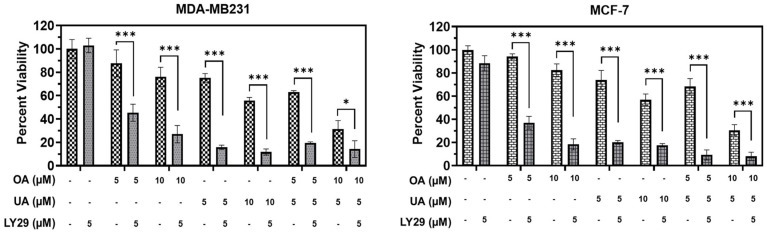
The PI3K inhibitor LY294002 enhances the cell death induced by pentacyclic triterpenes (PTTs). MTT assay-based cell viability was measured after treatment with OA, UA, and both with and without LY294002 (LY29); a specific inhibitor of PI3K) in both the BrCa cell lines. LY29 boosted the cytotoxicity induced by the PTTs. Results are expressed as means ± standard deviation (SD) (n = 3), and pairwise *t*-tests were used to determine significance: *p* < 0.05 = *, *p* < 0.001 = ***.

Most inducers of cytoprotective autophagy do so by inhibiting PI3K, a critical step in MTORC1 signaling. However, cytotoxic autophagy involves the use of drugs that inhibit PI3K as well. Usually, nontoxic PI3K inhibitors, such as LY294002, may help the cytotoxic drug induce a more robust additive or synergistic response. We found that co-incubation of LY294002 was not significantly cytotoxic to MCF7 or MDA-MB231 cells as measured by MTT assay (Figure 8); however, co-incubation with the TTPs significantly decreased cell viability at lower doses of both OA and UA in both BrCa cell lines (Figure 8). The dose–effect combined cytotoxicity of OA, UA, or OA + UA with 5 µM of LY294002 was synergistic.

## 4. Discussion

The data presented in this study establish that OA and UA have cytotoxic effects against human BrCa cells at significantly lower doses when combined. The combination treatment synergistically (combination index (C.I.) < 1.0) inhibited cell viability at ≤10 µM, including at a dose of 5 µM each. Based on our cell viability and colony-forming assays, we found that OA is less effective than UA. Still, these two PTTs are more effective in combination treatment, especially against MDA-MB231 (TNBC) cells (Figure 1B–E), which may point to their potential clinical application in BrCa patients with terminal stages of the disease. TNBC is the terminal stage of breast cancer for which only palliative treatments exist. Cytotoxic chemotherapy is predominantly administered, or a combination of chemoradiation and hyperthermia, or even combined chemoradiation therapy (“hot chemotherapy”), with a survival advantage of no more than a few months [39].

Our results showed that select PTTs were differentially toxic to tumorigenic cells, eliminated the clonogenic potential of treated cells at a reasonable dose (<7 µM), and did not kill non-tumorigenic breast epithelial cells that grew at a similar rate and in a similar growth medium. The cancer-specific toxicity of UA or OA + UA was more than 400% at UA 10 µM and OA + UA 5 + 5 µM, calculated from dose-equivalent toxicity between tumorigenic (MDA-MB231, MCF7) and non-tumorigenic (MCF-12A) cells.

In contrast to the shared mechanism of cell death induced by cytotoxic chemotherapy drugs used in BrCa therapy, such as doxorubicin, paclitaxel, and docetaxel, the PTTs did not kill BrCa cells by apoptotic mechanisms, as we were unable to identify any markers of apoptosis in PTT-treated cells (Figure 2A,B). Although when cells are treated with staurosporine, a positive inducer of apoptosis by its potent inhibition of several protein kinases [40], cleaved PARP bands and increased TUNEL signal showing DNA fragmentation were seen in both BrCa cell lines, indicating that apoptosis may not be a significant mechanism of cell death. This finding is supported by previous reports [14,41,42,43]. We could not find significant evidence for OA + UA-induced necroptosis or ferroptosis, as shown by the complete absence of any apoptosis markers in OA-, UA-, or OA + UA-treated cells (Figure 2). However, others have reported UA-induced apoptosis at higher concentrations (e.g., >30 µM) [42,44].

The other mechanism of the cell death pathway, predominantly autophagy (Figure 3A), was significant. The expression of LC3BII and P62/SQSTM1 (both molecular markers of autophagy) was high after OA and UA treatment, indicating that autophagy may be the primary mechanism of cell death in both ER+ and TNBC cells (MCF7 and MB231, respectively). Along with the expression of LC3BII, the formation of autolysosomes and induction of LC3B puncta are molecular indicators of autophagy [45]. As shown in Figure 3B, in both BrCa cell lines, LC3BII-containing puncta and autolysosome formation was high in UA-treated cells, indicating autophagy cell death because of UA or OA + UA treatment. This was further confirmed by increased LC3BII levels in cells simultaneously treated with UA and pepstatin A, a potent inhibitor of lysosomal protein degradation (Figure 3C) [14,41,42,43]. Previous studies have shown that the AKT/mTOR pathway is critical for the regulation of autophagy and site-specific phosphorylation/dephosphorylation of many proteins that are involved in autophagic pathways, such as ULK1 (Unc-51-like autophagy-activating kinase 1; homologue of ATG1 in mammals), P70S6 (ribosomal protein S6 kinase beta 1), 4EBP1 (eukaryotic translation initiation factor 4E-binding protein 1), etc., play critical roles in the execution of autophagy [46,47,48].

Autophagy-related proteins (ATGs) are essential for initiating phagophores, sequestration, autophagosome formation, and overall autophagy regulation. Approximately 40 types of ATGs have been identified that regulate or coordinate the formation of autophagosomes. Out of these, ATG5 and ATG7 are essential regulators of autophagy. We demonstrated their role in the PTT-induced execution of autophagy that progressed through ATG5/ATG7, which was reversed and increased the survival of tumor cell populations by silencing them [49]. As previously mentioned, the regulation of autophagy involves the AKT/mTOR pathways and the site-specific phosphorylation of AKT and mTOR. Treatment with OA and UA (in MDA-MB231 cells) significantly suppressed mTOR phosphorylation (as shown in Figure 4) and site-specific AKT phosphorylation in a time-dependent manner (shown in Figure 6) in both BrCa cell lines. Moreover, OA and UA reduced AKT phosphorylation at Thr308, associated with mTORC1 activation, and AKT phosphorylation at the Ser473 site, which plays a role in the phosphorylation and activation of mTORC2. Therefore, OA and UA treatment significantly decrease AKT’s activity by inhibiting both sites [50]. This inhibition, escorted by the decrease in phosphorylation of P70S6K and increase in phosphorylation of 4EBP1 (as shown in Figure 4), ultimately leads to mTORC1 and mTORC2 becoming inactivated, which leads to activation of autophagy [4,6].

OA and UA have shown activity in several cell systems at high doses (≥50 µM to 0.4 mM). UA has anticancer, antidiabetic, and antiobesity activities at 150–500 mg/kg/day [16,51]. Administration of UA in feed (1% *w*/*w*) for up to 36 weeks did not show toxicity and achieved a serum level of ≥3 µM [52]. In limited human studies, OA and UA have shown tolerable toxicity profiles at an oral dose of 0.45 g to 3 g once daily for up to 8 weeks [27]. However, none of these studies tested the efficacy of the OA + UA combination, although they are structural isomers and are likely to coexist in variable ratios among edible plants. A sole chemodietary combination of OA + UA by themselves or in a neoadjuvant and adjuvant setting for patients with TNBC may benefit them by increasing their life span in terms of disease-free survival or reduced disease severity.

## 5. Conclusions

In conclusion, this study demonstrates the therapeutic potential of OA and UA in breast cancers when used at low doses, and these are equally elective on both TNBC and ER+ BrCa, represented by MDA-MB231 and MCF7 cells, respectively. The results also demonstrate that the combination of OA + UA is more effective than UA alone, although OA at the tested dose alone did not promote cytotoxicity. The study provides valuable insights into the underlying mechanisms of PTT-induced cell death and highlights its future potential as a nontoxic adjuvant therapy for BrCa patients.

## Figures and Tables

**Table 1:** List of fine chemicals used in this study.

Fine Chemicals	CAS No.	Manufacturer and Catalogue No.
Oleanolic acid (OA)	508-02-1	TCI America (Portland, OR, USA) O0317
Ursolic acid (UA)	77-52-1	Sigma-Aldrich U6753
LY294002	154447-36-6	LC Laboratories (Woburn, MA, USA) L-7988
Rapamycin	53123-88-9	LC Laboratories R-5000
Staurosporine	62996-74-1	Fisher Scientific/MedChem Express (Monmouth Junction, NJ, USA) NC1401148
TUNEL assay kit		Roche (Indianapolis, IN, USA) 11684795910

**Table 2:** List of antibodies used in this study.

Name of Antibody	Manufacturer and Catalogue No.	RRID
PARP	Cell Signaling Technology (Danvers, MA, USA) 9542	AB_2160739
mTOR	Cell Signaling Technology 2983	AB_2105622
p-mTOR (Ser2481)	Cell Signaling Technology 2974	AB_2262884
LC3B	ABclonal (Woburn, MA, USA) A7198	AB_2863546
P62/SQSTM1	Cell Signaling Technology 5114	AB_10624872
ULK-1	Cell Signaling Technology 4773	AB_2288252
pULK-1 (Ser757)	Cell Signaling Technology 6888	AB_10829226
P70S6 Kinase	ABclonal A2190	AB_2749844
p-P70S6 Kinase (Ser371)	Cell Signaling Technology 9208	AB_330990
4EBP1	Cell Signaling Technology 9644	AB_2097841
p-4EBP1 (Thr37/46)	Cell Signaling Technology 2855	AB_560835
AKT (Pan)	Cell Signaling Technology 9272	AB_329827
p-AKT (Thr308)	Epitomics (Burlingame, CA, USA) 2214	
p-AKT (Ser473)	Cell Signaling Technology 4060	AB_2315049
ATG-5	Cell Signaling Technology 2630	AB_2062340
ATG-7	Cell Signaling Technology 2631	AB_2227783
β-Actin	Proteintech (Rosemont, IL, USA) HRP-60008	AB_2819183
Anti-rabbit Secondary	Cell Signaling Technology 7074	AB_2099233
Fluorescence-labeled Secondary Antibody	Goat anti-Rabbit IgG (H + L) Alexa Fluor™ 488 A11008	AB_143165

## Data Availability

Data are shown in the Section 3, along with Supplementary Materials details. Also, they are available upon request from the corresponding author.

## Supplementary Materials

The following supporting information can be downloaded at https://www.mdpi.com/article/10.3390/cancers16193367/s1. Figure S1: Isobologram analysis of UA- and OA-treated BrCa cells. File S1: Original images of western blots.
