# Supplementary material for: Cytotoxic Autophagy: A Novel Treatment Paradigm against Breast Cancer Using Oleanolic Acid and Ursolic Acid
Source: Cancers (Basel). 2024 Oct 1;16(19):3367. doi: 10.3390/cancers16193367 (PMC11476055; doi:10.3390/cancers16193367)
Supplement: Supplementary file 1 [file cancers-16-03367-s001.zip › cancers-3218562 - File S1.pdf]

Blots for Fig-2

PARP

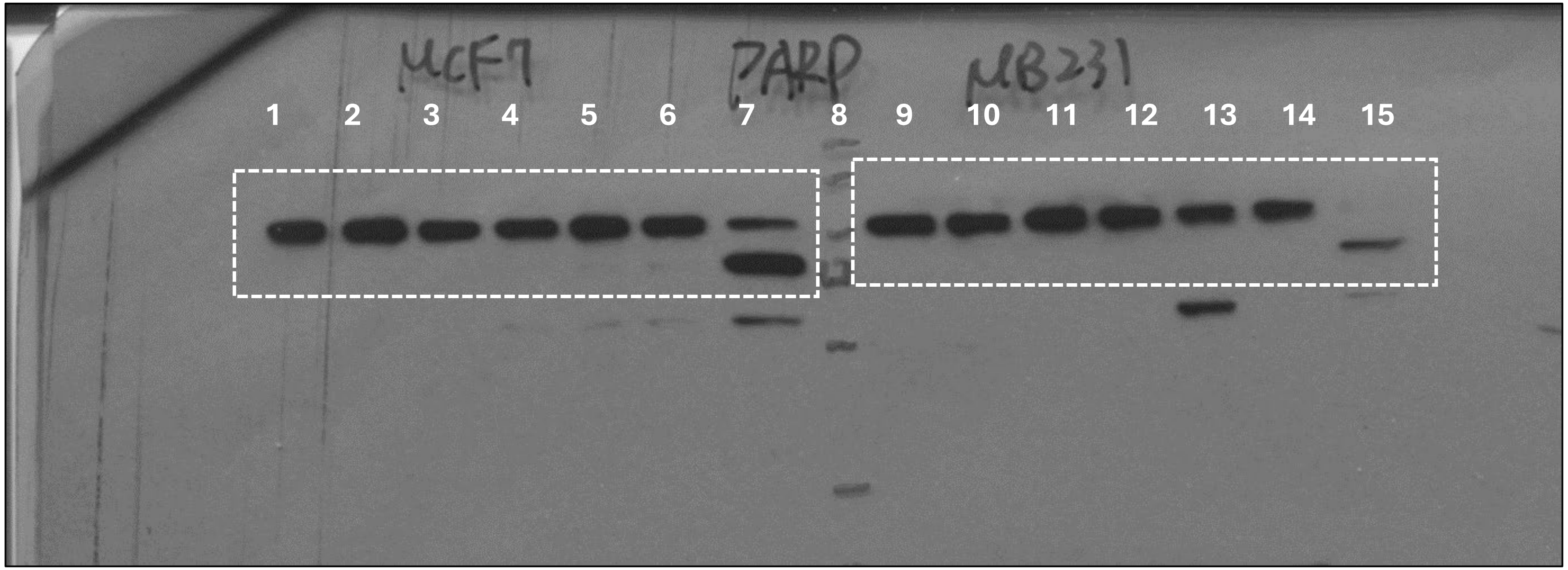

β-Actin

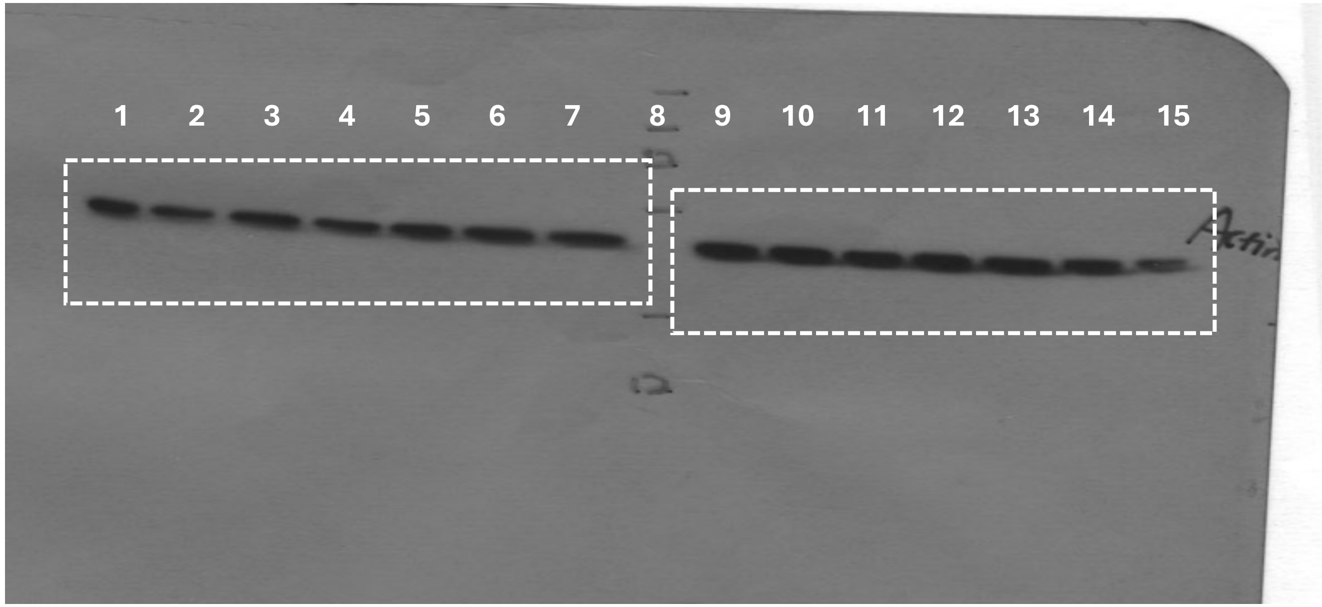

Lane No.	Sample ID	Cell Line
1	Vc	MCF7
2	5µM, OA	
3	10µM, OA	
4	5µM, UA	
5	5µM, OA + 5µM, UA	
6	10µM, OA + 5µM, UA	
7	2µM, Sta	
8	Protein Marker	
9	Vc	MDA-MB231
10	5µM, OA	
11	10µM, OA	
12	5µM, UA	
13	5µM, OA + 5µM, UA	
14	10µM, OA + 5µM, UA	
15	2µM, Sta	

Blots for Fig-3.A

Lane No.	Sample ID
1	Vc
2	5µM, OA
3	10µM, OA
4	5µM, UA
5	5µM, OA + 5µM, UA
6	10µM, OA + 5µM, UA
7	2µM, Rapa
8	Other Samples
9	Other Samples

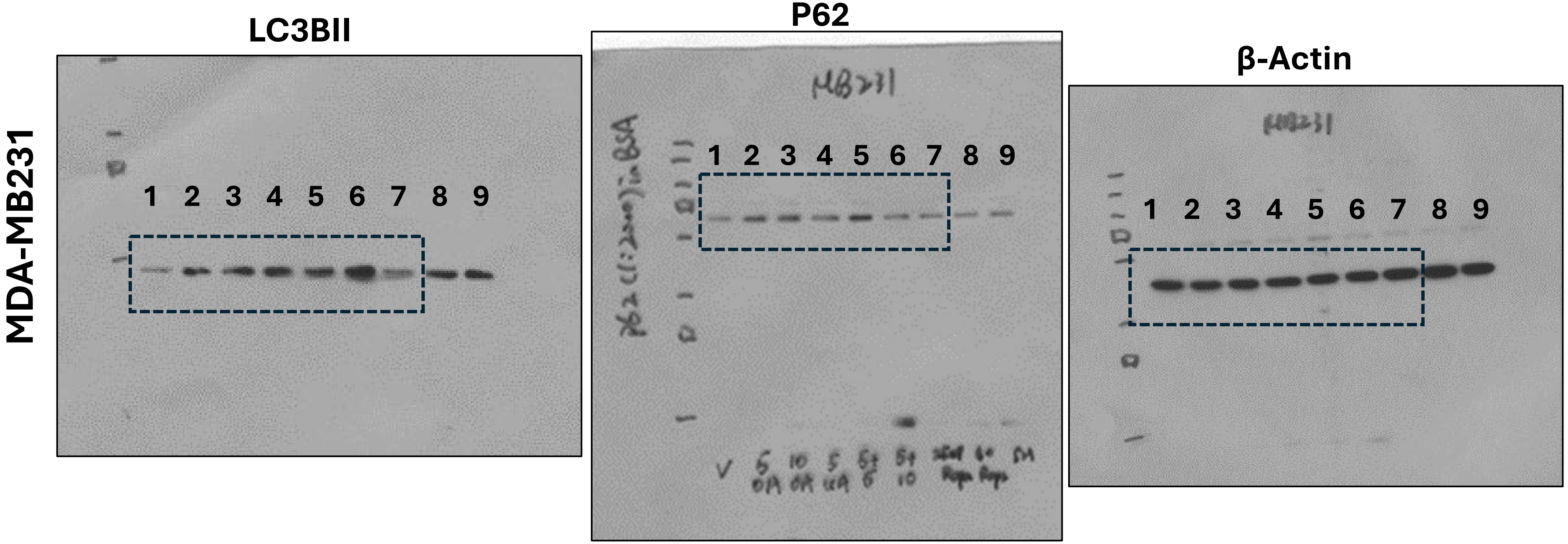

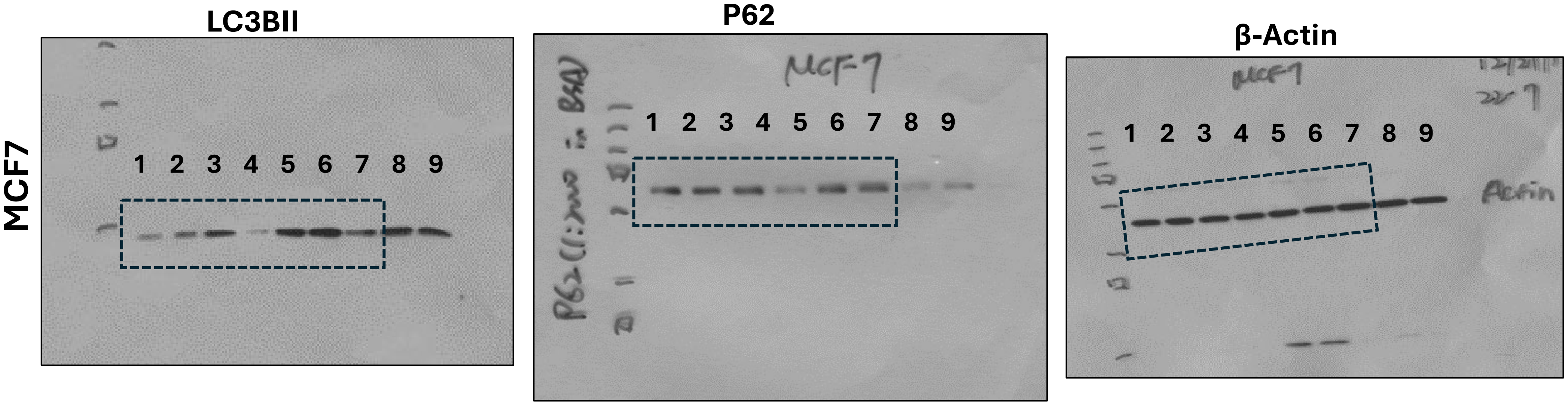

Blots for Fig-3.C

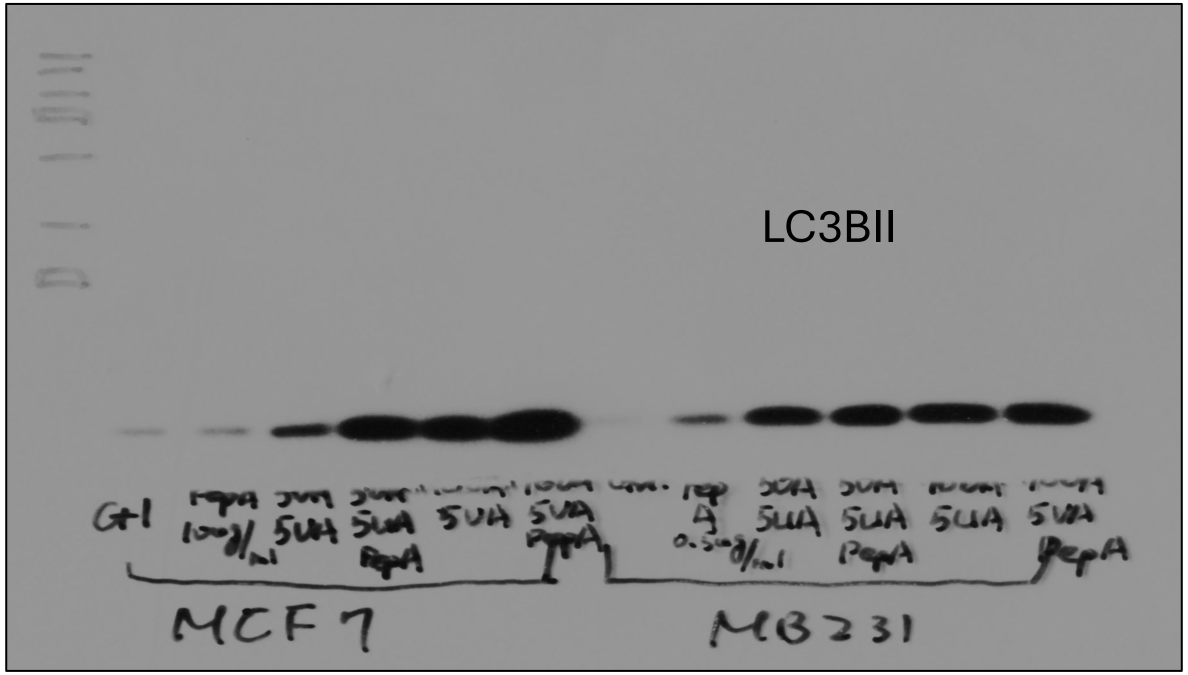

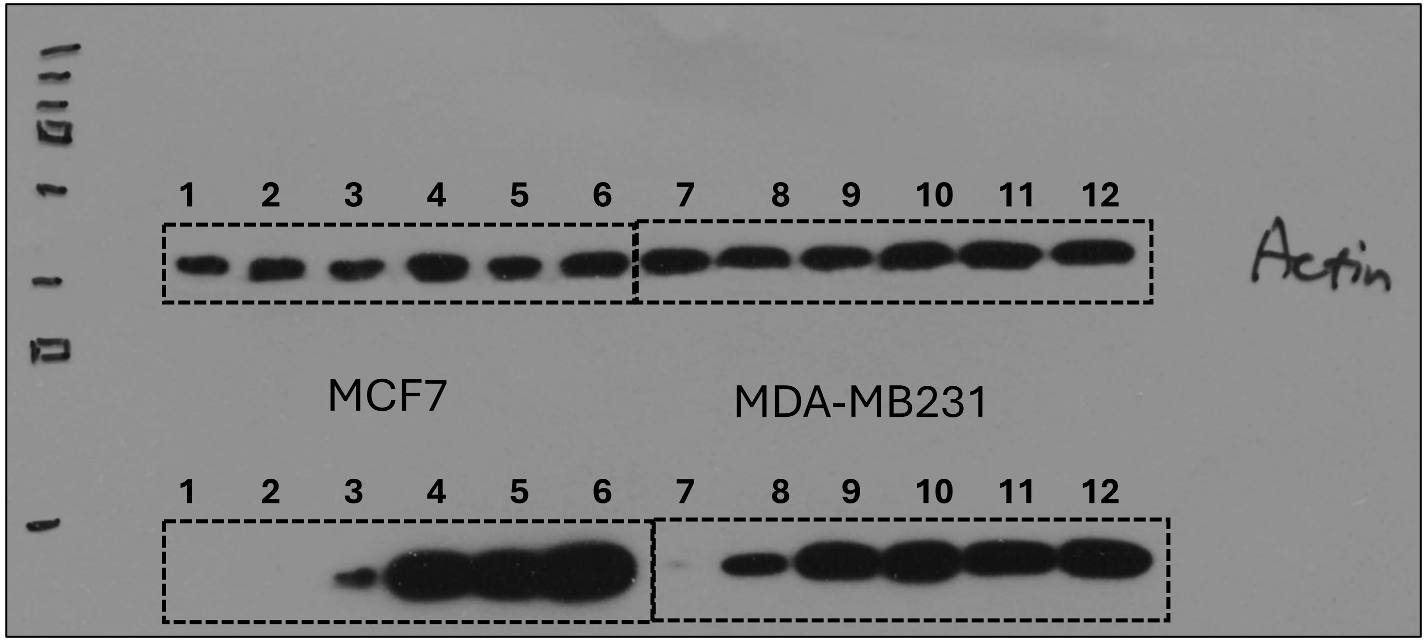

Lane. No.	Sample ID	Cell Lines
1	Untreated	MCF7
2	10µg/ml Pep-1	
3	5µM, OA + 5µM, UA	
4	5µM, OA + 5µM, UA + 10µg/ml Pep-1	
5	10µM, OA + 5µM, UA	
6	10µM, OA + 5µM, UA + 10µg/ml Pep-1	
7	Untreated	MDA-MB231
8	10µg/ml Pep-1	
9	5µM, OA + 5µM, UA	
10	5µM, OA + 5µM, UA + 10µg/ml Pep-1	
11	10µM, OA + 5µM, UA	
12	10µM, OA + 5µM, UA + 10µg/ml Pep-1	

Blots for Fig-4

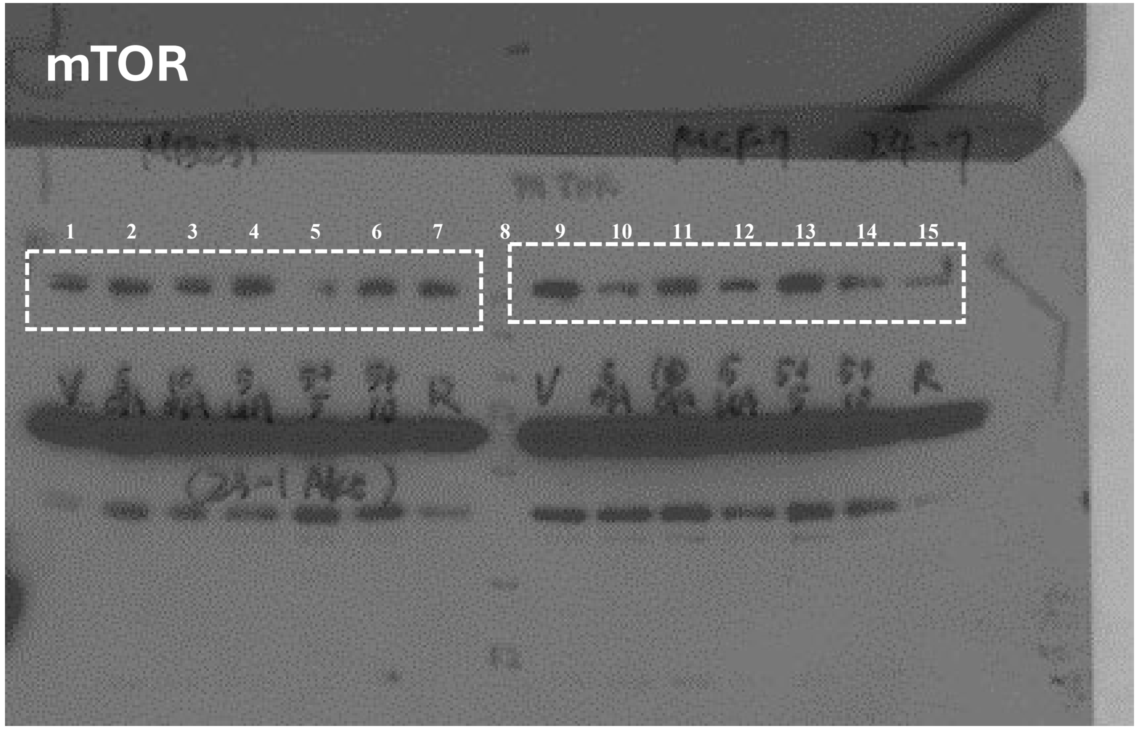

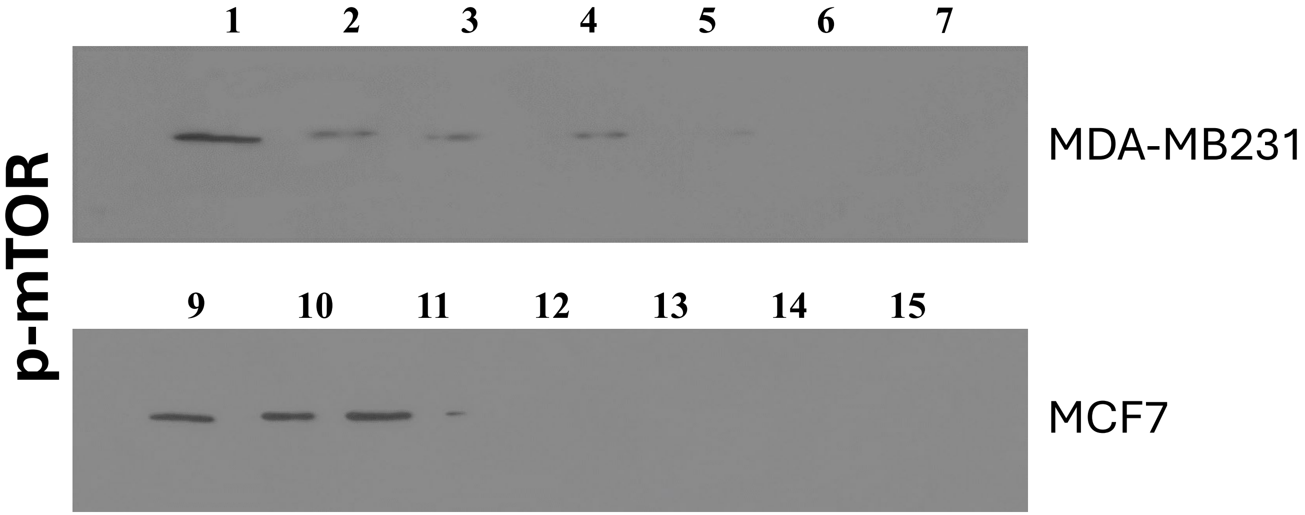

Lane No.	Sample ID	Cell Line
1	Vc	
2	5μM, OA	
3	10μM, OA	
4	5μM, UA	MDA-MB231
5	5μM, OA + 5μM, UA	
6	10μM, OA + 5μM, UA	
7	2μM, Rapa	
8	Protein Marker	
9	Vc	
10	5μM, OA	
11	10μM, OA	
12	5μM, UA	
13	5μM, OA + 5μM, UA	MCF7
14	10μM, OA + 5μM, UA	
15	2μM, Rapa	

Blots for Fig-4 Cont…

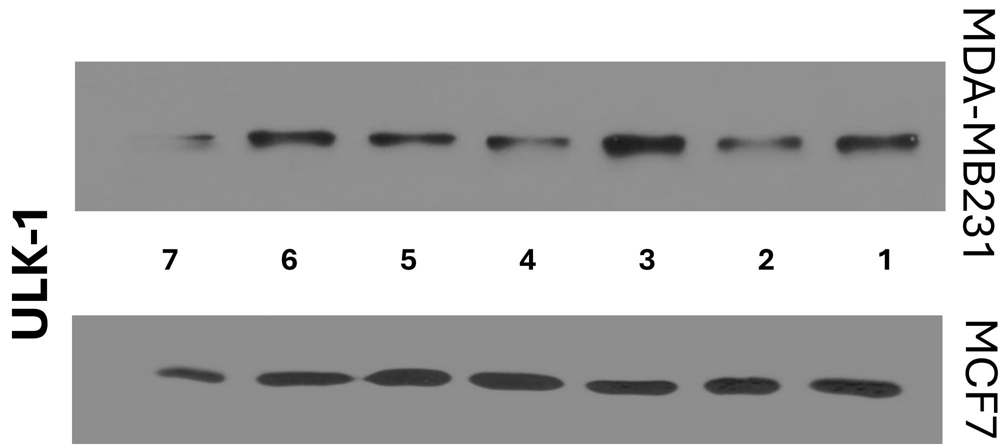

Lane. No.	Sample ID
1	2μM, Rapa
2	10μM, OA+ 5μM, UA
3	5μM, OA+ 5μM, UA
4	5μM, UA
5	10μM, OA
6	5μM, OA
7	Vc
8	Other test samples
9	Other test samples

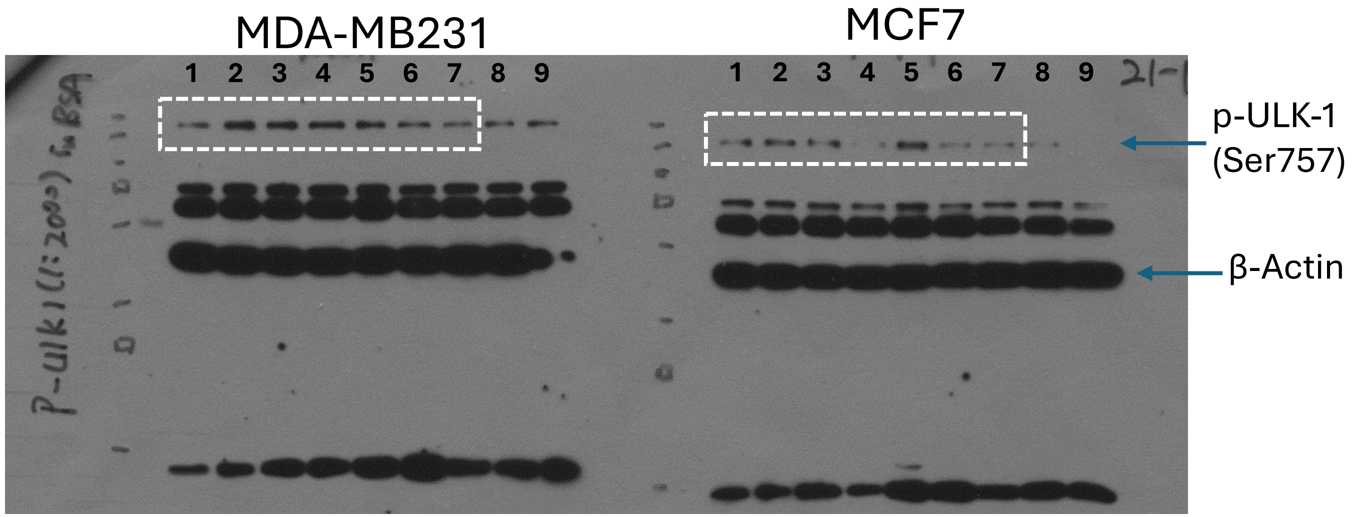

Blots for Fig-4 Cont...

MDA-MB231

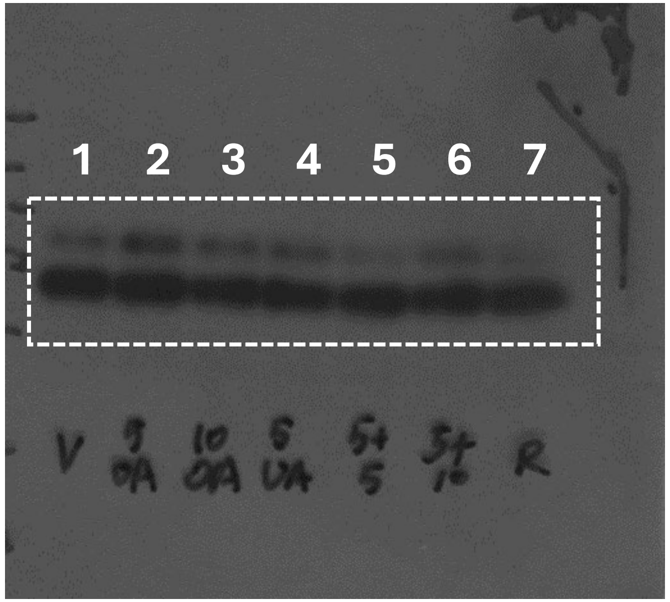

P70S6
kinase

MCF7

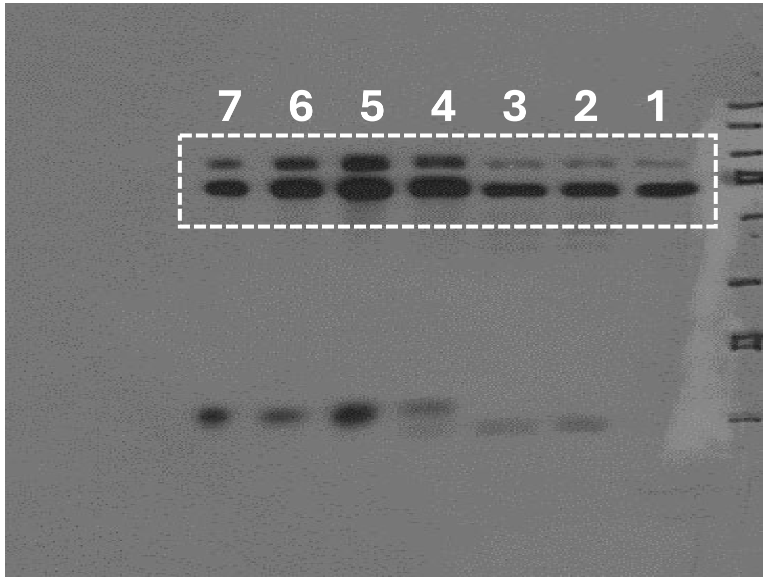

Lane No.	Sample ID
1	Vc
2	5µM, OA
3	10µM, OA
4	5µM, UA
5	5µM, OA<br>+ 5µM, UA
6	10µM, OA<br>+ 5µM, UA
7	2µM, Rapa

MDA-MB231

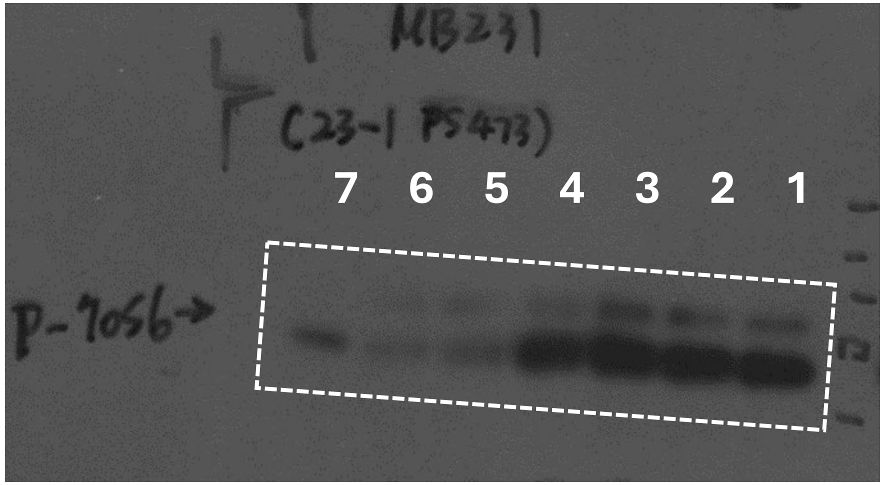

p-P70S6
(Ser371)
kinase

MCF7

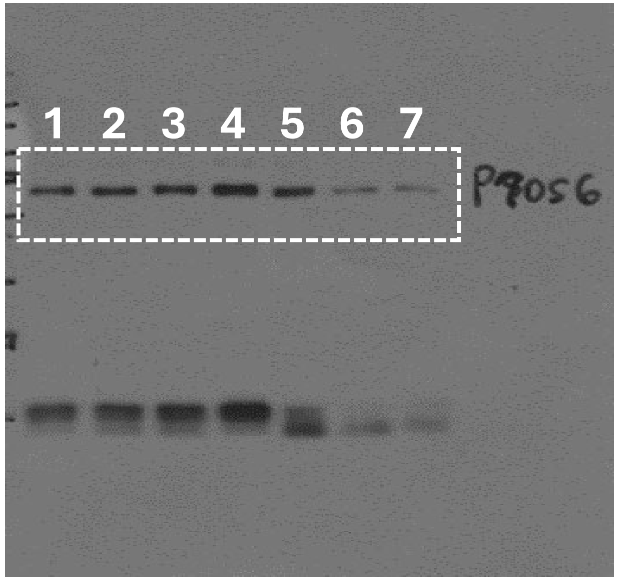

Blots for Fig-4 Cont...

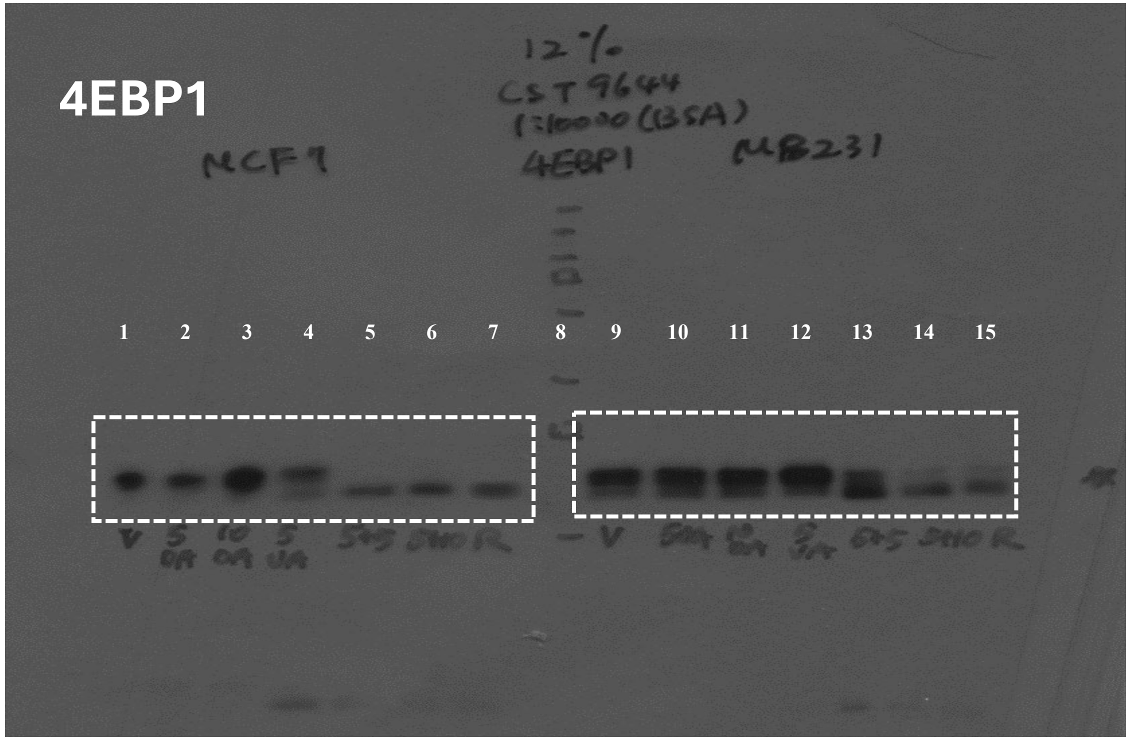

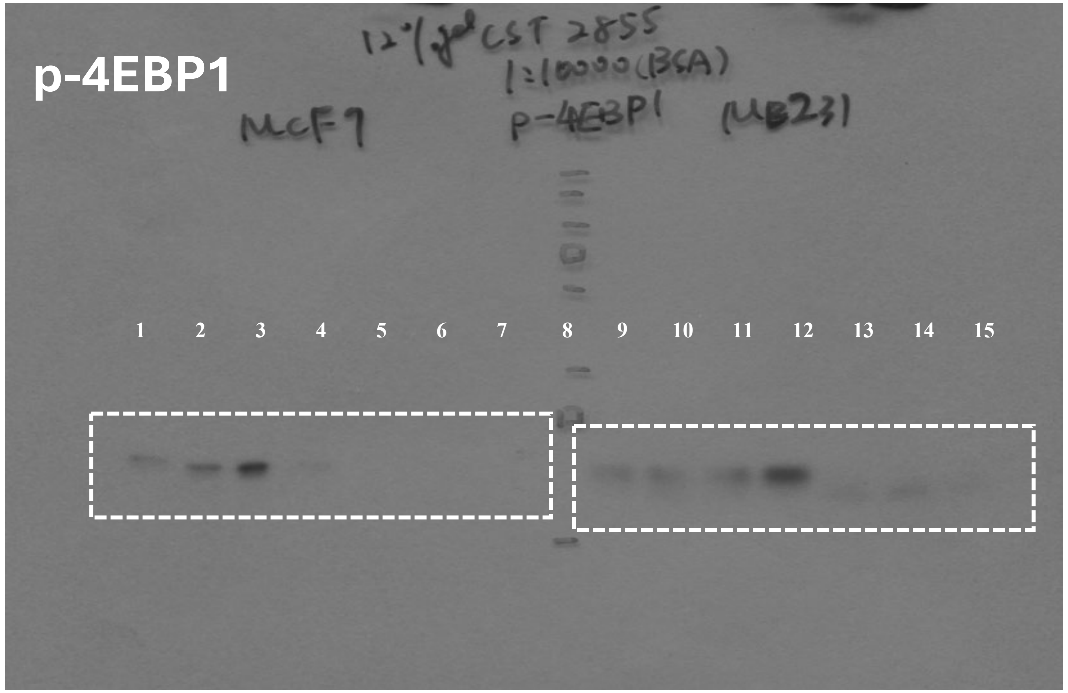

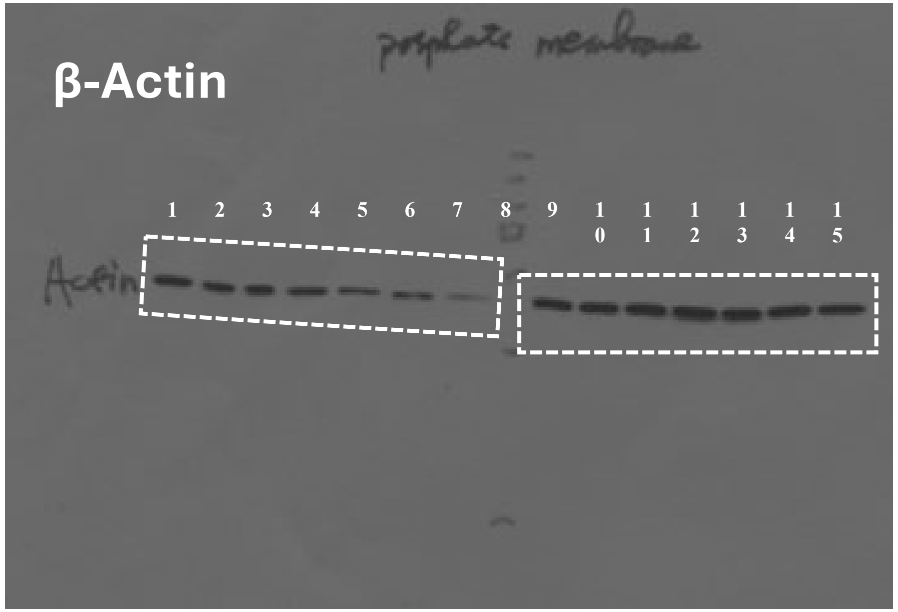

Lane No.	Sample ID	Cell Line
1	Vc	MCF7
2	5μM, OA	
3	10μM, OA	
4	5μM, UA	
5	5μM, OA + 5μM, UA	
6	10μM, OA + 5μM, UA	
7	2μM, Rapa	
8	Protein Marker	
9	Vc	MDA-MB231
10	5μM, OA	
11	10μM, OA	
12	5μM, UA	
13	5μM, OA + 5μM, UA	
14	10μM, OA + 5μM, UA	
15	2μM, Rapa	

Blots for Fig-5.A

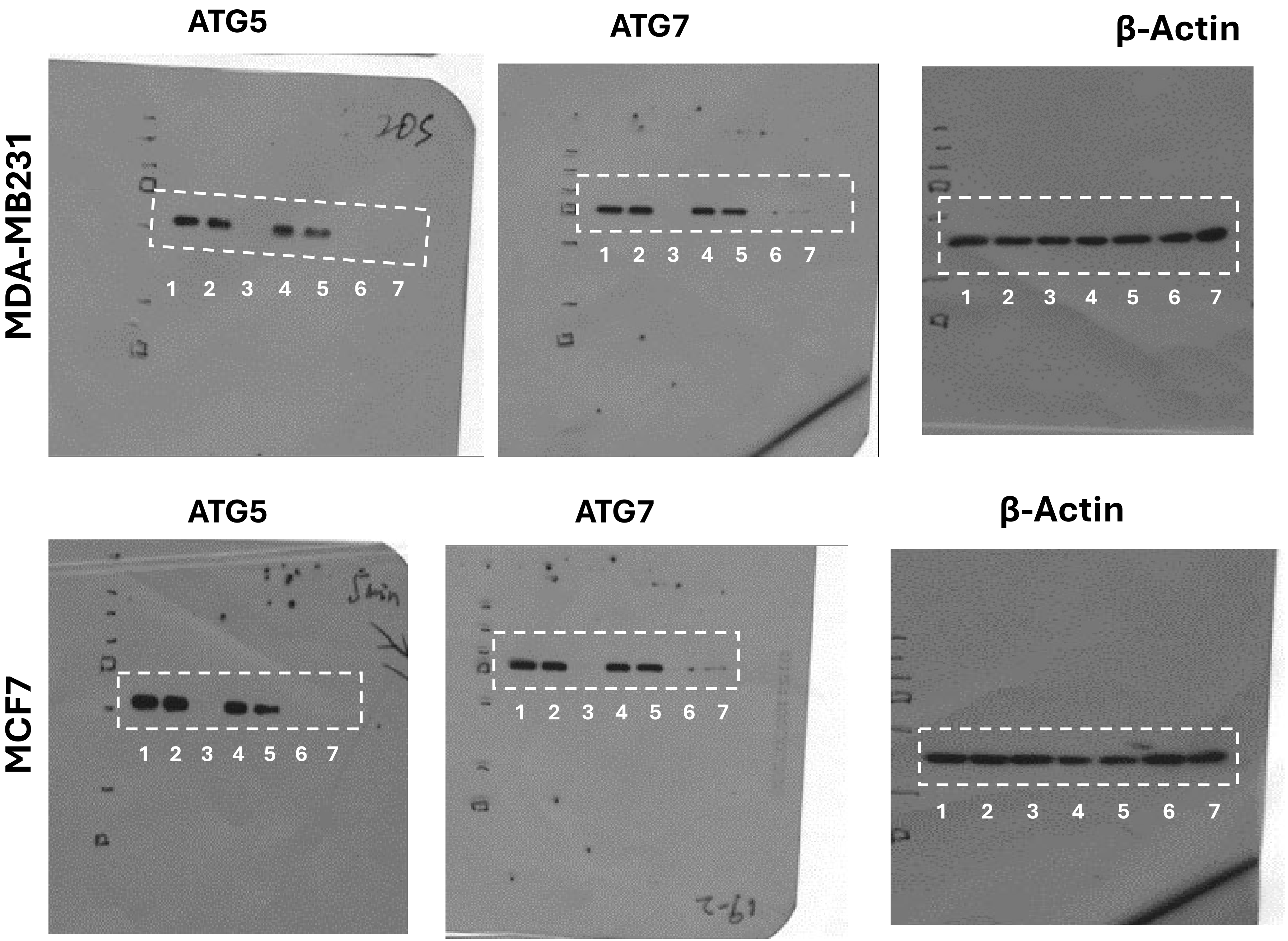

Lane No.	Sample ID
1	Untreated
2	Scrambled siRNA
3	ATG specific siRNA
4	5μM, OA + 5μM, UA
5	10μM, OA + 5μM, UA
6	Specific siRNA + 5μM, OA + 5μM, UA
7	Specific siRNA + 10μM, OA + 5μM, UA

Blots for Fig-5.B

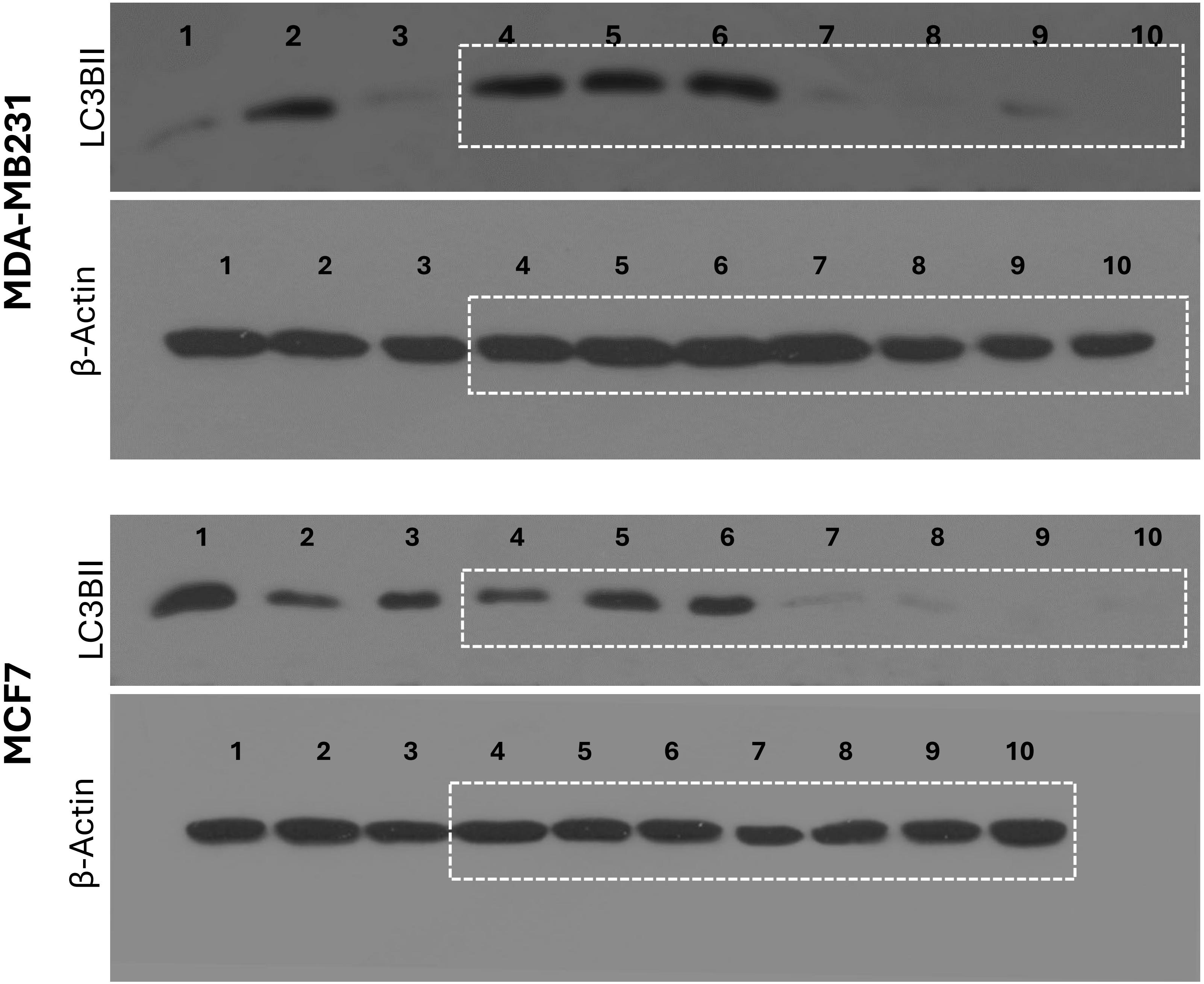

Lane. No.	Sample ID
1	Other Test Samples
2	Other Test Samples
3	Other Test Samples
4	Ctrl siRNA + 10μM, OA
5	Ctrl siRNA + 5μM, UA
6	Ctrl siRNA+ 10μM, OA + 5μM, UA
7	ATG-5 siRNA + 10μM, OA
8	ATG-5 siRNA + 5μM, UA
9	ATG-7 siRNA + 10μM, OA
10	ATG-7 siRNA + 5μM, UA

Blots for Fig-6

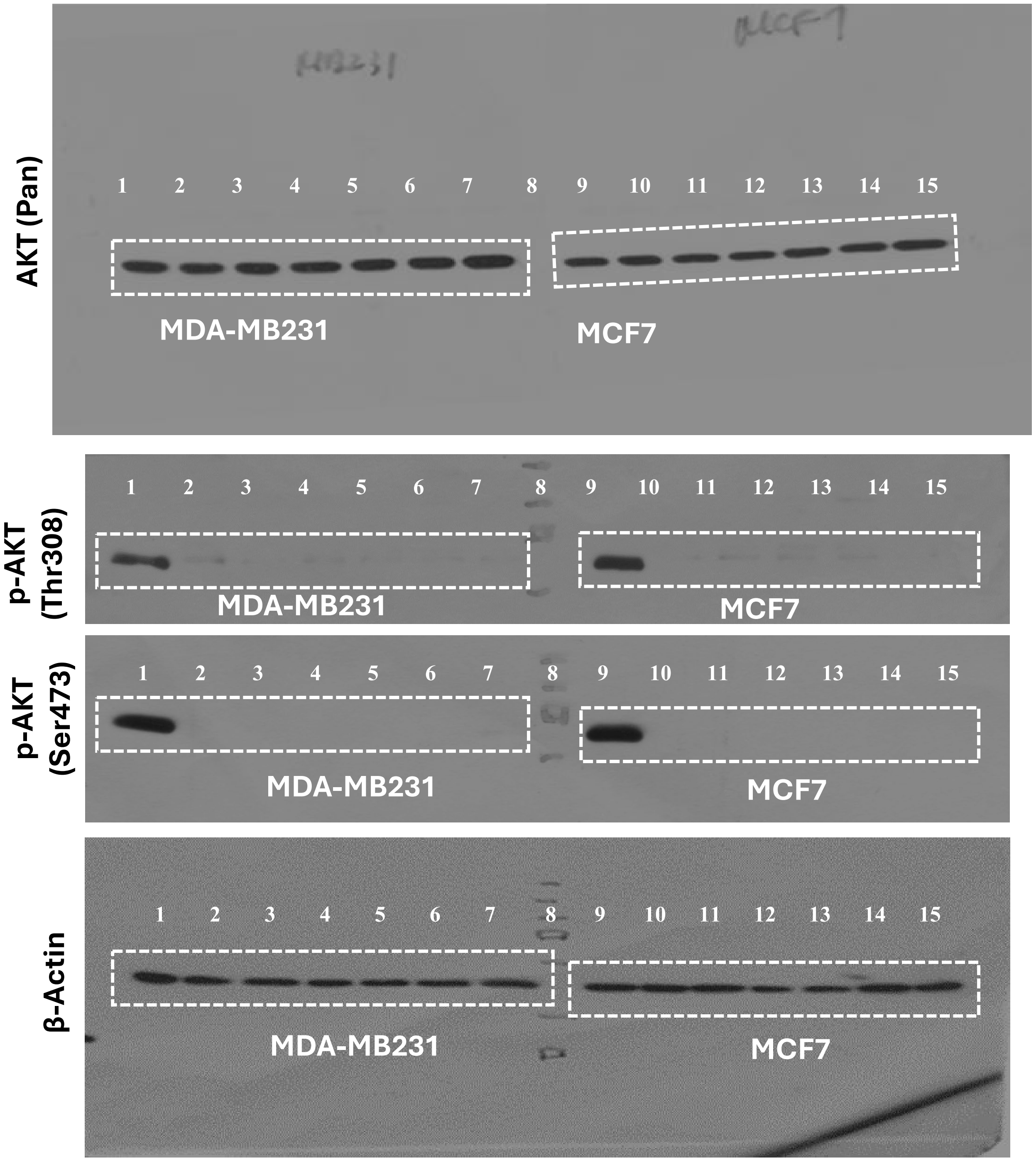

Lane No.	Sample ID	Cell Line
1	Vc	MDA-MB231
2	5μM, OA	
3	10μM, OA	
4	5μM, UA	
5	5μM, OA + 5μM, UA	
6	10μM, OA + 5μM, UA	
7	2μM, Rapa	
8	Protein Marker	
9	Vc	MCF7
10	5μM, OA	
11	10μM, OA	
12	5μM, UA	
13	5μM, OA + 5μM, UA	
14	10μM, OA + 5μM, UA	
15	2μM, Rapa	
