# Supplementary material for: Cytotoxic Autophagy: A Novel Treatment Paradigm against Breast Cancer Using Oleanolic Acid and Ursolic Acid
Source: Cancers (Basel). 2024 Oct 1;16(19):3367. doi: 10.3390/cancers16193367 (PMC11476055; doi:10.3390/cancers16193367)
Supplement: Supplementary file 1 [file cancers-16-03367-s001.zip › cancers-3218562 - Supplementary Figure S1.pdf]

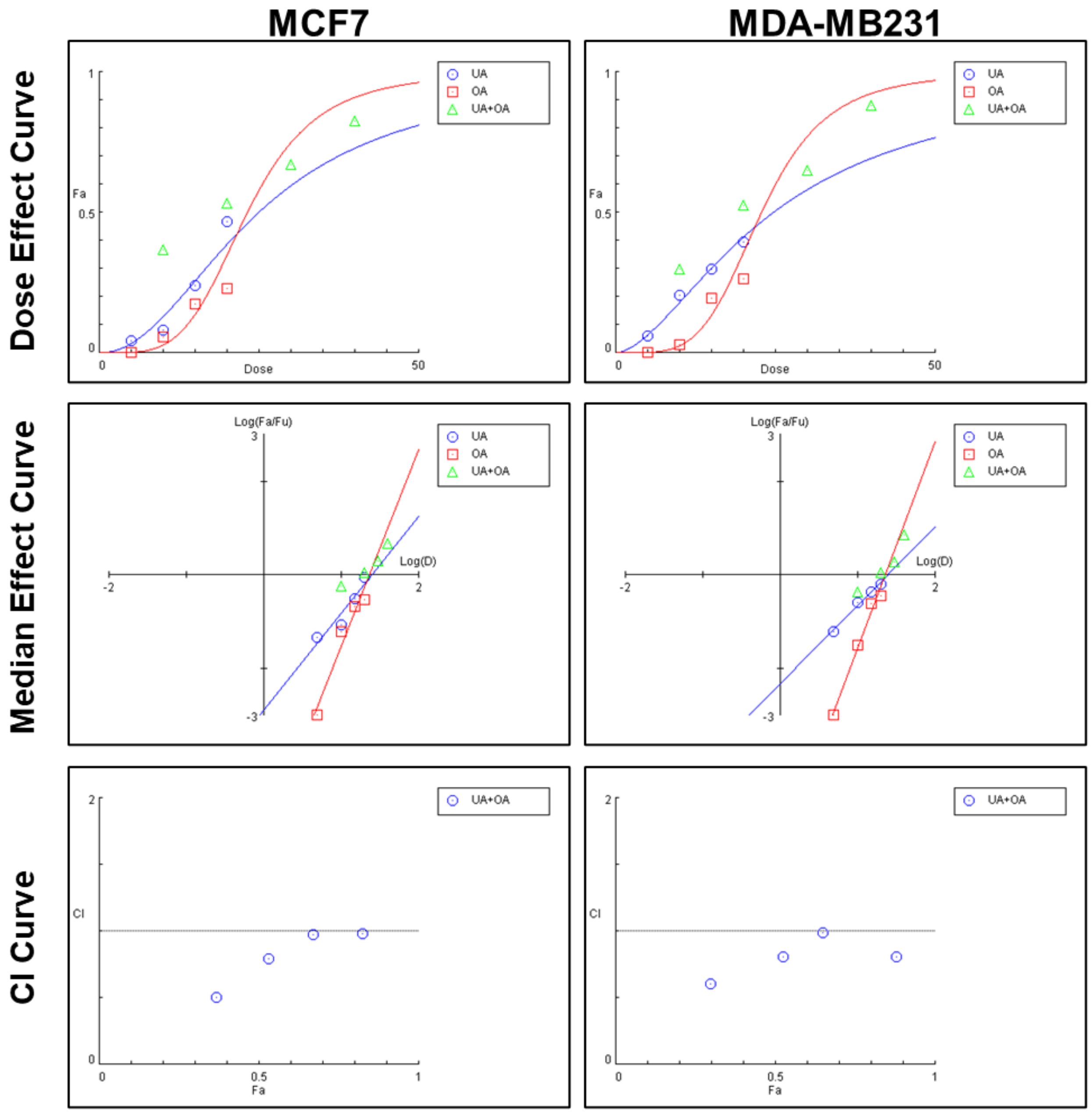

Combination Index Data					
Cell Type	Dose of UA (μM)	Dose of OA (μM)	Dose Effect	CI Values	Remark
MCF7	5	5	0.3682	0.50492	Synergism
	10	10	0.5328	0.79516	Synergism
	15	15	0.6714	0.97431	Synergism
	20	20	0.8244	0.98166	Synergism
MDA-MB231	5	5	0.2988	0.60224	Synergism
	10	10	0.5258	0.80953	Synergism
	15	15	0.6509	0.99124	Synergism
	20	20	0.8799	0.80762	Synergism
